# Hydrothermal carbonization as an alternative sanitation technology: process optimization and development of low-cost reactor

**DOI:** 10.12688/openreseurope.14306.2

**Published:** 2022-06-10

**Authors:** Jae Wook Chung, Gabriel Gerner, Ekaterina Ovsyannikova, Alexander Treichler, Urs Baier, Judy Libra, Rolf Krebs

**Affiliations:** 1 Institute of Natural Resource Sciences, Zurich University of Applied Sciences, Wädenswil, 8820, Switzerland; 2 Institute of Agricultural Engineering, University of Hohenheim, Stuttgart, 70599, Germany; 3 Institute of Chemistry and Biotechnology, Zurich University of Applied Sciences, Wädenswil, 8820, Switzerland; 4 Postharvest Technology, Leibniz Institute for Agricultural Engineering and Bioeconomy, Potsdam-Bornim, 14469, Germany

**Keywords:** hydrothermal carbonization, sanitation, energy, nutrient, faecal sludge, resource recovery, struvite, fertilizer

## Abstract

**Background:** The provision of safe sanitation services is essential for human well-being and environmental integrity, but it is often lacking in less developed communities with insufficient financial and technical resources. Hydrothermal carbonization (HTC) has been suggested as an alternative sanitation technology, producing value-added products from faecal waste. We evaluated the HTC technology for raw human waste treatment in terms of resource recovery. In addition, we constructed and tested a low-cost HTC reactor for its technical feasibility.

**Methods: **Raw human faeces were hydrothermally treated in a mild severity range (≤ 200 °C and ≤ 1 hr). The total energy recovery was analysed from the energy input, higher heating value (HHV) of hydrochar and biomethane potential of process water. The nutrient contents were recovered through struvite precipitation employing process water and acid leachate from hydrochar ash. A bench-scale low-cost reactor (BLR) was developed using widely available materials and tested for human faeces treatment.

**Results: **The hydrochar had HHVs (23.2 - 25.2 MJ/kg) comparable to bituminous coal. The calorific value of hydrochar accounted for more than 90% of the total energy recovery. Around 78% of phosphorus in feedstock was retained in hydrochar ash, while 15% was in process water. 72% of the initial phosphorus can be recovered as struvite when deficient Mg and NH _4_ are supplemented. The experiments with BLR showed stable operation for faecal waste treatment with an energy efficiency comparable to a commercial reactor system.

**Conclusions:** This research presents a proof of concept for the hydrothermal treatment of faecal waste as an alternative sanitation technology, by providing a quantitative evaluation of the resource recovery of energy and nutrients. The experiments with the BLR demonstrate the technical feasibility of the low-cost reactor and support its further development on a larger scale to reach practical implementation.

## Plain language summary

In less developed places where modern wastewater treatment systems are unavailable, relatively simple onsite technologies are implemented for human waste treatment. Faecal sludge produced from those facilities needs proper treatment to ensure public health and resource recovery. In this research, we studied raw human faeces treatment with hydrothermal carbonization technology, which converts wet biomass into a charcoal-like solid material (hydrochar) and organic-rich liquid (process water) at relatively mild temperature (~200 °C) under pressurized conditions (~20 bar). We tested several reaction parameters to find an optimum point for the best energy recovery. The total energy value of the resulting materials was analysed by measuring the heating value of hydrochar and biogas production from process water. The remaining nutrient contents in hydrochar ash and process water was recovered through struvite precipitation which can be used as a slow-releasing fertiliser. Hydrochar derived from human faeces showed comparable heating values to bituminous coal. Around 70% of initial phosphorus in faeces was recovered as struvite with supplements of magnesium and ammonium from external sources. Also, we developed a bench-scale low-cost reactor using ordinary pipe-fitting materials and tested it for faecal waste treatment. These results present the initial evidence for technical feasibility and resource recovery. Further research for testing various faecal sludges in field conditions and developing pilot-scale low-cost reactors are encouraged.

## Introduction

Untreated human excreta pose serious risks to public health and environment. In 2020 almost half of the global population still does not have access to safely managed sanitation services that properly contain and treat the faecal waste. In the least developed countries (LDCs), the conditions are most severe with only 26% of people covered by suitable services. To be able to achieve the Sustainable Development Goal (SDG) aimed at universal access to safely managed sanitation services by 2030, the current rate of progress in LDCs must be accelerated 15 times ( WHO & UNICEF, 2021). 

In the places where centralised sewer collection and wastewater treatment systems are unaffordable or inefficient, onsite or decentralised technologies such as pit latrines and septic tanks can be implemented as the collection method. The faecal sludge generated from these technologies requires appropriate service chains that target the safe end-use or disposal of the final product ( Strande *et al*., 2014). Since one of the main reasons for insufficient service provision is the high economic burden placed on stakeholders, the development of profitable business opportunities could lead to breakthroughs in this sector ( Boot & Scott, 2009). Although various options for final product valorisation have been proposed, including fuel briquette, biogas generation and protein feed (Black soldier fly larvae), the most common practice being implemented is agricultural application through composting which also has the lowest economic value of the options ( Mallory *et al*., 2020).

In recent years, hydrothermal carbonization (HTC) has been investigated to treat faecal waste for sanitisation and resource recovery. It is a thermochemical process that converts wet biomass into more carbonaceous materials at relatively low temperatures (~200 °C) under pressurized conditions (~20 bar) which results from autogenic pressure development. Since HTC treats wet feedstocks, it has the advantage of lower energy requirements compared to conventional dry pyrolysis technologies that need coupling with an energy-intensive drying process ( Libra *et al*., 2011). The output from the HTC treatment usually has the form of a thick slurry which further can be separated into solid hydrochar (HC) and organic-rich process water (PW). These products have a wide range of potential applications e.g. as energy sources, soil amendment, fertiliser, and adsorbent ( Sharma *et al*., 2020). Hydrothermal treatment of different types of faecal wastes has produced HC materials with enhanced higher heating values (HHV) ranging from 16 – 28 MJ / kg, using primary sewage sludge ( Danso-Boateng *et al*., 2013), raw human faeces ( Wüst *et al*., 2019; Yahav Spitzer *et al*., 2018), faecal sludge ( Fakkaew *et al*., 2018) and their simulants ( Afolabi *et al*., 2017). In general, applying higher temperatures and longer reaction times has been found to increase the HHV in HC materials but with a lower solid yield. In addition to improving the fuel value of faecal waste, nutrients can be recovered from the HTC products. Wüst *et al*. (2019) reported that a considerable proportion of phosphorus and nitrogen in raw human waste was retained in the solid phase after the HTC treatment, while alkali salts were dissolved in PW. Especially the recovery of phosphorus, an essential nutrient for living organisms, can be an important advantage in LDC sanitation services, since it is a limited resource globally ( Cordell *et al*., 2009; Cordell *et al*., 2011) and in many local agricultural applications. Diminishing high-quality phosphorus sources and the exploitation of low-quality sources with higher transport, extraction and processing costs cause a greater cost burden on farmers ( Desmidt *et al*., 2015). The inclusion of a technology in the local sanitation service chain that can aid in the reutilisation of phosphorus in waste streams for local food production may lead to the required breakthroughs in this sector.

Despite the strong potential of HTC as a sanitation technology, there is still not enough research pursuing its implementation in less developed communities with limited technical and economic resources. Since the major cost factors of HTC technology arise from reactor construction and energy consumption for treatment, optimizing operational parameters in a low-temperature range would be beneficial in lowering both capital and operating costs ( Song *et al*., 2019). Previous research has highlighted the potential for cost reduction in HTC reactor construction. A batch type reactor with 20 L capacity was constructed employing a relatively simple design and function at a material cost of around EUR 9 K (in 2013, excluding personnel and certification cost), which was several times cheaper than a commercial reactor system with comparable treatment capacity ( Robbiani, 2013).

The purpose of this research was to investigate HTC technology as an alternative sanitation technology for faecal waste treatment, mainly focussing on the technical feasibility of the low-cost approach and its potential for resource recovery of energy and nutrients. Firstly, raw human waste was treated in a HTC commercial reactor at a variety of mild operational conditions. The total energy recovery was evaluated based on the energy input to the process and recovery through the products of both phases. The resulting materials from the best case were analysed for phosphorus recovery through the struvite precipitation process. Secondly, a low-cost reactor employing ordinary pipe-fitting materials and a simple heating method was constructed and validated for faecal waste treatment using the selected operational conditions. Based on these results, a scenario for energy and nutrient recovery through hydrothermal treatment of faecal waste is discussed.

## Methods

All experimental data was processed by Microsoft Excel (2018).

### Human waste feedstock and hydrochar

Two stocks of human faeces were collected at a composting toilet company (Kompotoi, Zurich, Switzerland) and processed in separate batches. Ethical approval was not required for this study because the materials were collected from toilets used by anonymous users. First the larger additives, toilet paper and woody cover materials, were sorted out from the raw stock. The collected material was then autoclaved at 121 °C for 20 min and dried in an oven at 105 °C overnight. The dry matter (DM) content of the stock was around 25%. The remaining smaller additives other than faecal mass was removed from the dry stocks, before being ground in a mechanical grinder (GM 300, Retsch, Haan, Germany) and stored at 4 °C until used. The first (Faeces-1) and the second (Faeces-2) stocks were used for optimization experiments and BLR experiments, respectively. The feedstock for each HTC run was prepared by mixing the designed amount of dry faeces with demineralised water to a solids content of 20% DM (cf. semi-solid faecal sludge has 15–25% DM)( Velkushanova *et al.*, 2021). Then, this mixture was placed at room temperature overnight to ensure sufficient reassociation of dry faeces and water. After the HTC, the resulting slurry from the HTC experiment was separated by either vacuum filtration employing filter paper with a pore size of 11 µm (Whatman, Maidstone, UK) or centrifugation (5910R, Eppendorf, Hamburg, Germany) at 4300 relative centrifugal force (RCF) for 3 min. The HC was dried in an oven at 105 °C overnight. The resulting HC and PW samples were stored at 4 °C until used. Solid samples for chemical analyses were further powdered by a ball mill (MM400, Retsch GmbH, Haan, Germany).

### HTC procedure and reactors

Two sets of HTC experiments for faecal waste treatment were carried out. Firstly, selected operational parameters were tested with a commercial reactor system to find the condition for best energy recovery ratio (energy content / energy input). Then, a bench-scale low-cost reactor (BLR) was developed and tested for HTC conditions obtained from the previous optimization experiments.

***Process optimization in commercial batch reactor.*** In the optimization study, several process parameters were tested (at 160 °C for 10 and 60 min, at 180 °C for 10 and 60 min, and at 200 °C for 1 s, 10 and 60 min) to find an optimum point for best energy recovery ratio using a commercial batch reactor system (Kiloclave, Büchi AG, Uster, Switzerland). Briefly, 480g feedstock (Faeces-1) was hydrothermally treated in a pressure vessel with an active volume around 640 mL (vessel volume subtracted by the volume of in-vessel equipment e.g. stirring rod, baffle, and stem of temperature probe) at an approximate fill-up rate of 3 / 4. The reactor jacket was equipped with a temperature sensor and an electric heating mantle of a 1000 W capacity. The temperature (TR) and pressure inside the reactor and the throttle of the heating mantle were monitored in a 20-seconds interval. The heating intensity was regulated by a proportional–integral–derivative (PID) controller as programmed for each test condition. To approximate the conditions expected in low-cost reactor systems, stirring and active cooling after the reaction were not applied. Two operational procedures were followed: 1) the reactor was heated to the target temperature and held there for a holding time (10 or 60 minutes), or 2) the heating was stopped immediately when the target temperature was reached, called Touch & Down (TD). This new operational regime was tested to simulate the conditions where automated temperature control is unavailable.

In total, seven experimental conditions were tested in duplicate ( Table 1) for HTC process optimization. The Test ID indicates the reactor temperature and the holding time e.g. Test-160-10 for HTC at 160 °C for 10 minutes and Test-200-60 for HTC at 200 °C for 60 minutes. The TD case, where the heating stopped when TR = 200 °C, is named Test-200-0. Only one TD case was studied, since the number of runs was limited by the amount of available feedstock. The choice of 200 °C was based on the results from preceding optimization experiments, in which the lower temperature range (160-180 °C) resulted in insufficient dewaterability of the resulting slurry from HTC treatment.

**Table 1.** Features of hydrothermal carbonization tests and characteristics of resulting materials. Results from hydrothermal carbonization experiments.

† ‡Test ID	Temp. (°C)	Time (min)	*Severity Factor (SF)	Time at Target Temp. ± 3 % (min)	**Adj. SF	Peak Pressure (bar)	pH	Ultimate analysis and ash contents of Hydrochar (%)						Hydrochar Yield (%)	Process Water (g)
								C	H	N	#S	§O	Ash		
Faeces-1								49.6	6.7	4.2	0.5	28.8	10.2		
Faeces-2								48.8	7.4	4.5		28.4	10.9		
160-10	160	10	2.77	15.9 ± 0.6	2.97	5.9	5.7	51.6	7.4	3.6	0.4	27.6	9.4	76.0 ± 0.7	270.1 ± 1.3
160-60		60	3.54	72.5 ± 3.5	3.63	5.9	5.5	52.6	7.5	3.4	0.5	26.1	9.9	71.3 ± 0.5	261.7 ± 5.4
180-10	180	10	3.36	17 ± 1.0	3.58	10.1	5.5	52.9	7.5	3.4	0.4	25.9	9.9	70.7 ± 0.7	272.5 ± 8.6
180-60		60	4.13	71.9 ± 0.9	4.21	10.7	5.2	54.7	7.6	3.2	0.4	23.8	10.3	67.7 ± 0.3	279.1 ± 3.6
200-0	200	1 s	0.02	4.3	3.58	16.1	5.3	54.5	7.7	3.2	0.5	23.9	10.2	69.9 ± 0.2	284.0 ± 5.0
200-10		10	3.94	20.9 ± 0.2	4.26	16.7	5.2	55.3	7.6	3.2	0.4	22.5	11.0	67.6 ± 0.1	282.7 ± 5.1
200-60		60	4.72	73 ± 2.3	4.80	19.8	5.2	56.4	7.6	3.1	0.4	21.7	10.8	65.7 ± 0.7	279.7 ± 13.1
BLR-160-10	160	10	2.77	24	3.14	7.3	5.9	52.2	7.6	3.8		25.4	11.0	70.7	883.0
BLR-200-0	200	1 s	0.02	12	4.02	21.0	5.7	55.0	7.5	3.7		21.7	12.1	65.5	890.8

† Tests performed with the commercial reactor system: n=2, and results from composite samples prepared by combining resulting materials from both runs. The values indicate the average of two observations ± difference to individual data points. ‡ Tests performed with the BLR: n=1. * Severity factor, Heidari *et al*., 2019. ** Adjusted severity factor based on the duration at set temperature ± 3 %. # Sulfur contents obtained from ICP-OES analysis § Oxygen contents estimated by subtracting the CHNS and ash fraction from 100 %.

The reaction severity factor (SF) of each condition was presented according to the Equation (1) and Equation (2) ( Heidari *et al*., 2019). Also, an adjusted severity factor (SF _adj_) was obtained by substituting t with t _adj_ in the Equation (1) for better comparison of TD cases with other tests. 


$$R\;=\;t\;e^{\frac{\left(T-100\right)}{14.75}}\;(1)$$

SF=log⁡R(2)

where R is the reaction ordinate (min); t is the reaction time (min); T is the reaction temperature (°C); t _adj_ is the time interval in which the reactor actually stays inside the range of ±3% of for the set temperature (min).

***Bench-scale low-cost reactor.*** The BLR was constructed by a pressure vessel manufacturing company (Bero Technik, Sirnach, Switzerland). Details of the designing consideration, technical drawing and parts information are available as *Extended data* ( Chung, 2021). Briefly, a 2 L vessel was fabricated using austenitic stainless steel pipe and fitting materials (EU code - 1.4404 / US - 316L) through circumferential welding technique. The main reactor body consists of a blind flange, weld-neck flange, pipe and end cap. It was equipped with a bimetal temperature gauge, a bourdon tube pressure gauge and a bleed valve. For safety assurance, an overpressure valve (set at 25 bar) and a rupture disc were installed. The BLR was mounted on a heating unit obtained / modified from previous research ( Bleuler *et al*., 2021). A portable camping stove with a 3000 W capacity (Easy Fuel Duo, Primus, Stockholm, Sweden) was used to heat the BLR. The weight of the fuel gas bottle was measured before and after the HTC to estimate energy input.

Two HTC conditions chosen from the optimization experiments were performed in the BLR in singlicate, Test-160-10 and Test-200-0. Hereafter, these tests were noted as BLR-160-10 and BLR-200-0, respectively. For each run, 1.5 kg of faecal feedstock (Faeces-2) with a solids content of 20% DM filled approximately 3 / 4 of the reactor volume. TR and pressure inside the reactor were monitored by gauge reading. The surface temperatures of the blind flange and gauge heads were measured using an infrared temperature gun (Fluke, Everett, US) with black insulation tape applied to the measurement surfaces. The pressure and temperature values were monitored in 3 min intervals. The heating intensity was controlled manually by adjusting the valve to the gas stove.

### Material characterization

The pH of the resulting slurry from HTC was measured with a pH meter (HQ40d, Hach, Loveland, US). Ash contents of HC and faeces samples were obtained according to DIN EN 14775 (heated at 550 °C for 120 min), using a muffle furnace (L 40 / 11 BO, Nabertherm, GmbH, Lilienthal, Germany). The resulting HC ash was used for subsequent experiments for nutrient recovery. Volatile solid (VS) of PW samples were measured according to 2540-E (liquid sample evaporated and dried at 105 °C overnight, then heated at 550 °C for 120 min). CHN compositions of solid samples were measured by a CHN analyser (TruSpec CHN, Leco, St. Joseph, US). Elemental analysis for PW, HC and HC ash was performed by the ICP-OES technique (Agilent 715, Agilent, Santa Clara, US) as previously reported ( Ovsyannikova *et al*., 2020). The partitioning of nutrient contents of the feedstock in resulting materials was calculated based on the mass (HC, PW and HC ash) obtained and the results from ICP-OES analyses. NH _4_-N concentration of PW was measured by the Hach-Lange cuvette test (LCK 304, Hach, Loveland, US).

### Energy analysis

The HHV of HC and dry faeces were measured with a bomb calorimeter (C200, IKA, Staufen, Germany). Biomethane potential (BMP) of the PW samples were analysed in batch tests according to VDI-4630 ( VDI, 2006) using an AMPTS system (BPC Instruments, Lund, Sweden). Briefly, microbial inoculum (anaerobic sludge) was collected at a wastewater treatment plant (ARA Rietliau, Wädenswil, Switzerland) and stabilised at room temperature for three days. 500 mL Schott bottles were filled with 390 g of inoculum and 10 g of the PW (substrate). The ratio of volatile solid of substrate (VSS) to volatile solid of inoculum (VSI) was around 0.11 for all PW bottles (VSS / VSI < 0.5 is recommended by VDI-4630). These bottles were purged with nitrogen gas for 3 min and placed in water baths set at 37 °C. The gas generated from bottles passed through 3 M NaOH solution supplemented with thymolphthalein (pH indicator - uncolored when pH < 9.3-10.5) to remove CO _2 _from the raw biogas, and the rest was considered CH _4_ ( Konrad *et al.*, 2021). Whole experimental procedures were validated by testing positive control (seeded with standard cellulose substrate) and blank (inoculum-only) samples. All substrates and blank tests were triplicated, and the average values were used for further calculation.

The process energy input for the experiments with the commercial reactor was estimated by an integration of the duration-throttle of the heating mantle, and that of BLR was obtained according to the model previously suggested by Danso-Boateng *et al*. (2015) with minor modifications as follows ( 3).


$$\overset{\begin{array}{l} \text{(Energy}\;\text{input)}\;\text{=}\;\text{(Energy}\;\text{to}\;\text{heat}\;\text{reactor)}\;\text{+}\;\text{(Energy}\;\text{to}\;\text{heat}\;\text{dry}\;\text{faeces)}\\ \text{+}\;\text{(Energy}\;\text{to}\;\text{heat}\;\text{water)}\;\text{+}\;\text{(Heat}\;\text{of}\;\text{reaction)}\;\text{+}\;\text{(Heat}\;\text{loss)}\;\text{(3)} \end{array}}{\underset{\begin{array}{l} {}[({\text{M}}_{\text{g0}})-{\text{M}}_{\text{g}})*\text{HV}]\;\text{=}\;{\text{[M}}_{\text{r}}{\text{*C}}_{\text{pr}}\text{*(T}-{\text{T}}_{\text{0}}\text{)]}\;\text{+}\;{\text{[M}}_{\text{f}}*{\text{C}}_{\text{pf}}*(\text{T}-{\text{T}}_{\text{0}})]\;\\ \;\;+\;[{\text{M}}_{\text{w}}*({\text{H}}_{\text{T}}-{\text{H}}_{\text{T0}})]\;+\;[{\text{M}}_{\text{0}}*\text{ΔHR}]\;+\;(\text{Heat}\;\text{loss})\;(4) \end{array}}{------------------------------------------------------------------------}}$$

where M _g0_ is the initial mass of fuel gas bottle (kg); M _g _ is the final mass of fuel gas bottle after HTC run (kg); HV is the HHV of fuel gas, estimated from its composition (49.4 MJ kg ^-1^); M _r_ is the reactor mass measured (21.5 kg); C _pr_ is the specific heat capacity of reactor material (0.5 kJ kg ^-1^ K ^-1^); T _0_ and T are the initial temperature and the target temperature (K), respectively; M _f_ = M _0 _is the mass of dry faeces in the feeding slurry (kg); C _pf _is the specific heat capacity of dry sewage sludge as a simulant for faeces (1.7 kJ kg ^-1^ K ^-1^) ( Namioka *et al*., 2008) ; M _w_ is the mass of water in feeding slurry; H _T0_ is the enthalpy of water at T _0_; H _T_ is the enthalpy of saturated water at T; ∆HR is the heat of reaction reported for 4 h reaction of faecal sludge (- 0.20 MJ kg ^-1^ at 160 °C and - 0.70 MJ kg ^-1^ at 200 °C) ( Danso-Boateng *et al*., 2015). The heat loss was calculated by difference. Finally, the energy recovery ratio for the HTC process is defined as the ratio of energy content to energy input. The energy content of HC (HHV of HC * mass of HC) and PW (HHV of methane * mass of biomethane produced from PW) was considered.

### Nutrient recovery through struvite precipitation

For the HTC condition yielded the best energy balance in optimization experiments (Test-160-10), recovery of phosphorus from hydrochar ash through struvite precipitation was investigated. Experimental procedures of previous research ( Ovsyannikova *et al*., 2020; Ovsyannikova *et al*., 2021) were adopted with minor modifications. To extract the phosphorus content from ash, 0.9 g of HC ash was added to 10 mL 1M H _2_SO _4_ to have the molar ratio between H ^+^ and P to 4:1 ( Petzet *et al*., 2012) and stirred overnight. The struvite precipitation was performed using 2 mL of the leachate with the supplement of PW (as NH _4_-N source) and MgCl _2_.6H _2_O (as Mg source) at designed doses for establishing the same molar ratio among P, NH _4_-N and Mg in the precipitation batch. For struvite precipitation, the pH of the solution was adjusted to 8 by the addition of 1M NaOH. The solution was stirred at 500 rpm during the pH setting and reaction time of 45 min. The precipitate formed was recovered by filtration through the filter paper (5–13 µm, VWR, Ulm, Germany) and dried at 35°C. The ICP-OES and CHN analyses were performed to investigate its composition. Struvite structure in the precipitate was verified by XRD technique using a D8 Advance (Bruker, Billerica, US) employing Cu radiation through Ni-filter. The K _α2_ component was numerically subtracted. The diffraction pattern was identified with the database of International Centre for Diffraction Data (ICDD). 

## Results

### HTC of faeces

All HTC treatments in this study showed distinctive transformation of the faeces when compared to the untreated raw material ( Chung *et al*., 2021). The odour associated with faeces was replaced with a smell like spent coffee grounds. The brown colour of feedstocks changed into dark brown-black in the end products. All resulting slurries except for the ones from Test-160-10 had good dewaterability and were vacuum-filtered through 11 µm pores. Due to its inferior dewaterability, the slurry from Test-160-10 was separated into solid and liquid phases by centrifugation. Table 1 summarizes the general features of HTC experiments and results of ultimate analyses of HC samples. The HC yields and changes in the major elemental composition of HCs showed dependency on the calculated SF values. Less HC was recovered from experiments with higher SFs, while the HC obtained at higher SFs had improved characteristics as solid fuel, i.e. they had higher carbon and lower oxygen contents. The adjusted SF was seen to be a more reliable indicator of the reaction characteristics when comparing TD cases with limited holding time at the target temperature, i.e. Test-200-0 and BLR-200-0, to non-TD cases.

**Figure 1.**
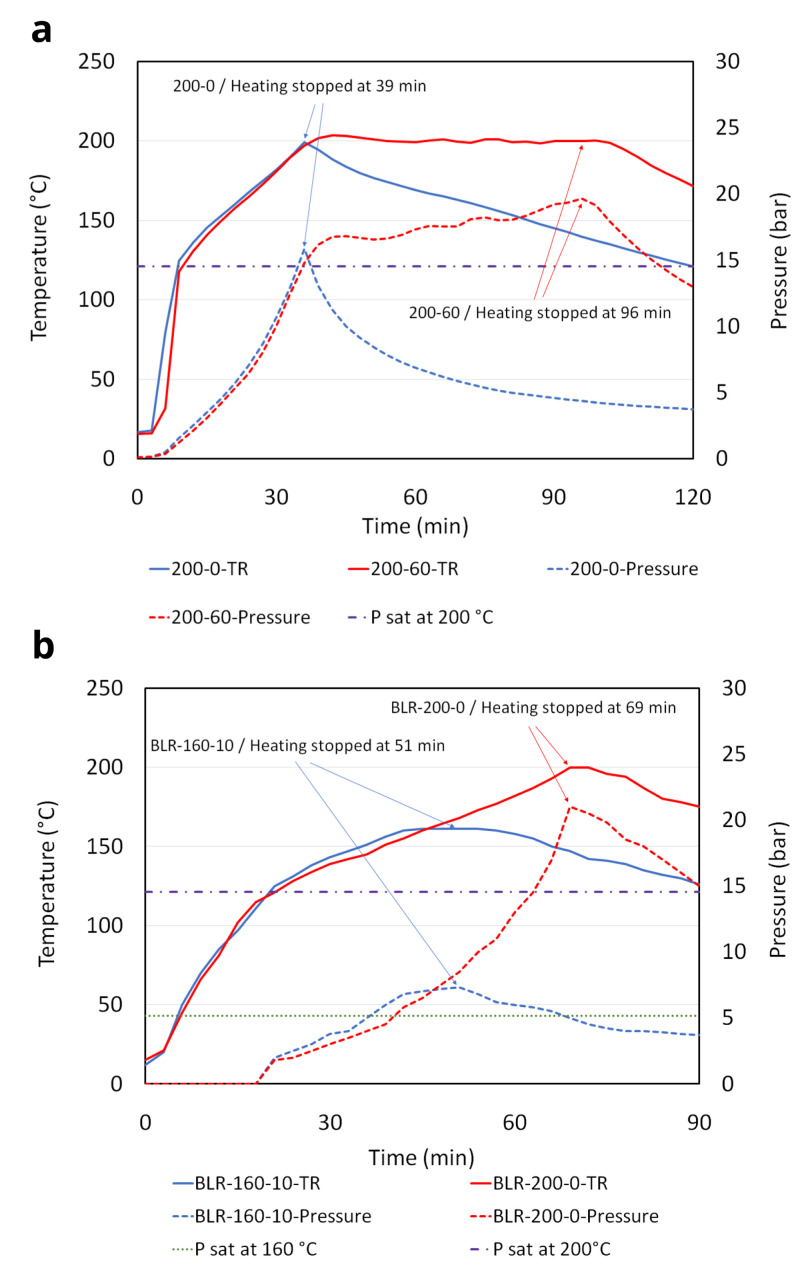
Temperature and pressure development during hydrothermal carbonization (HTC). ( **a**) Test-200-0 and 200-60 performed with the commercial reactor, ( **b**) Test-200-0 and 160-10 performed with the bench-scale low-cost reactor.

The peak pressure during HTC was primarily determined by the water vapor saturation pressure (Psat) which increased with TR. Substantial pressure development over Psat occurred for longer holding times and higher temperatures. Figure 1 shows the TR and pressure development from a few selected tests. The pressure increase over the holding time is visible when comparing the TD run with 60-minute holding, Test-200-0 vs. 200-60 in the commercial reactor ( Figure 1a). After reaching the target temperature of 200 °C, the pressure increased continuously above Psat as heating was applied to maintain the TR at the target temperature. The same phenomenon, but to a lower extent, was observed in the comparison between Test-180-10 and 180-60, while Test-160-10 and 160-60 did not show a significant difference in their peak pressure. This indicates that the hydrothermal conversion of the feedstock is taking place with gas generation during the holding time. Compared to the commercial reactor, the BLR had a lower heating rate and took almost twice as long to reach the target temperature 200 °C ( Figure 1b). Due to this slower rate, the TD run in the BLR (BLR-200-0) remained at temperatures above 180 °C much longer than the TD run in the commercial reactor (Test-200-0). Much more gas was produced in BLR-200-0 than in Test-200-0. This resulted in a higher peak pressure than the one obtained from the same test condition performed with the commercial reactor. Also, it was noteworthy in terms of safety assurance that the pressure development around the target temperature of 200°C in BLR was vigorous and approached the maximum operational pressure of 25 bar. In all cases, the pressure inside the reactor started to decrease immediately when the heating stopped, even while the TR was still greater than 180 °C. Throughout all experiments, the surface temperature of pressure and temperature gauge heads installed at BLR were below their allowable ambient temperature limit of 60 °C and 70 °C, respectively.

### Energy contents

The energy input for the process test conditions and energy content of the resulting materials are presented in Table 2. The combination of these two values in the energy recovery ratio (energy content / energy input) provides an indicator to compare the overall energy efficiency for the process and products of both phases. Looking first at HC; the HTC experiments with higher reaction severity densified the energy content in the solid phase to a greater extent, resulting in HHV values ranging between 23.2 and 25.2 kJ/g. In contrast, as seen in Table 1, the trend for the HC yield is inversely related to SF _adj_. Therefore, despite the differences in HHV, the energy content of HC was similar for all test conditions, ranging from 1.59 to 1.69 MJ. For PW, the energy content was determined in BMP experiments and also varies only slightly, between 0.08 and 0.13 MJ. In all cases, more than 90% of the biogas was generated within the first five days (data not shown), showing good potential for recovery. However, only 4 – 7% of the total energy input was recovered from the PW; the majority of the energy was recovered in the HC. Because the energy content from HC and PW were comparable for all test conditions employed in this research, the energy recovery ratio was primarily determined by the energy input. Since more energy input is required at the higher SF _adj_ conditions, this leads to lower energy recovery ratios as temperature and holding time are increased. While the Test-160-10 yielded more energy than what was invested, the Test-200-60 recovered only 52.4% of its energy input. Compared to the energy content of the feedstock, 75 – 80% and 4 – 6% of initial energy in faecal biomass were transformed into a form of solid fuel (HC) and biogas (PW), respectively ( Table 2).

**Table 2.** Energy input for test conditions and recovery from resulting materials. HTC=hydrothermal carbonization.

Test ID	Energy input during HTC (MJ)	Hydrochar (dry basis)				Process water					† Total ratio to E. input / †† Total ratio to E. content of feedstock (%)
		Weight (g)	HHV (kJ/g, dry basis)	Energy content (MJ)	Ratio to E. input / Ratio to E. content of feedstock (%)	Weight (g)	VS (g / g PW)	* CH 4 yield (g CH 4 / g VS)	Energy content (MJ)	Ratio to E. input / Ratio to E. content of feedstock (%)	
160-10	1.59 ± 0.02	72.9 ± 0.7	23.2	1.69 ± 0.02	106.4 / 80.9	270.1 ± 1.3	0.051	0.13 ± 0.04	0.09	5.5 / 4.3	111.9 / 85.2
160-60	2.35 ± 0.05	71.3 ± 0.5	23.7	1.69 ± 0.01	71.9 / 80.9	261.7 ± 5.4	0.054	0.11 ± 0.01	0.08	3.4 / 3.8	75.4 / 84.7
180-10	2.10 ± 0.03	67.7 ± 0.8	23.7	1.61 ± 0.02	76.7 / 77.0	272.5 ± 8.6	0.057	0.13 ± 0.01	0.10	4.7 / 4.8	81.4 / 81.8
180-60	2.81 ± 0.11	64.9 ± 0.3	24.6	1.60 ± 0.01	57.1 / 76.6	279.1 ± 3.6	0.054	0.15 ± 0.00	0.11	4.0 / 5.3	61.1 / 81.9
200-0	2.21 ± 0.09	67.0 ± 0.2	24.5	1.65 ± 0.00	74.6 / 78.9	284.0 ± 5.0	0.056	0.15 ± 0.03	0.13	5.8 / 6.2	80.4 / 85.1
200-10	2.41 ± 0.05	64.8 ± 0.0	25.0	1.62 ± 0.00	67.2 / 77.5	282.7 ± 5.1	0.052	0.18 ± 0.05	0.10	4.2 / 4.8	71.4 / 82.3
200-60	3.28 ± 0.05	63.1 ± 0.7	25.2	1.59 ± 0.02	48.7 / 76.1	279.7 ± 13.1	0.047	0.16 ± 0.03	0.12	3.7 / 5.7	52.4 / 81.8
BLR-160-10	4.80	212.0	23.2	4.93	102.6 / 76.8	883.0	0.048	not available			102.6 + / 76.8 +
BLR-200-0	6.14	196.6	24.4	4.80	78.2 / 74.8	890.8	0.047				78.2 + / 74.8 +
Faeces-1	96 g in feedstock		21.8	2.09		not applicable					
Faeces-2	300 g in feedstock		21.4	6.42							

- Average value ± difference to individual data points n=2 or * standard deviation n=3. - † (energy content of HC + PW) / energy input. - †† (energy content of HC + PW) / initial energy content of feedstock.

The experiments performed with BLR showed a better energy efficiency (i.e. energy consumption for carrying out the same task) than the commercial reactor employed in optimization experiments. To treat 1 kg dry faeces, the commercial reactor required 16.6 MJ for Test-160-10 and 23.0 MJ for Test-200-0. The values from BLR for the same test condition were 16.0 MJ for BLR-160-10 and 20.5 MJ for BLR-200-0. The energy input for two tests performed with the BLR showed a similar distribution between the energy uses ( Table 3). About half of the energy was used to heat the reactor and feedstock, and the other half was lost during the treatment as heat loss. The exothermic energy from the hydrothermal reaction of feedstock contributed to the total energy demand to a limited extent (< 4% of energy input). 

**Table 3.** Energy assessment for bench-scale low-cost reactor operation.

Contents	BLR-160-10	BLR-200-0
	MJ (% of energy input)	
Energy input	4.80 (100.0)	6.14 (100.0)
Energy to heat reactor	1.59 (33.1)	1.99 (32.4)
Energy to heat faeces	0.08 (1.6)	0.09 (1.5)
Energy to heat water	0.75 (15.6)	0.95 (15.4)
Heat of reaction	- 0.06 (1.3)	- 0.21 (3.4)
Heat loss	2.44 (50.9)	3.32 (54.1)

### Nutrient contents

The overall inorganic content of the solids as indicated by the ash content was hardly changed by the HTC treatment. The range remained similar to that of the dry faeces, 9.4–11.0 wt % ( Table 1). However, the trends of the individual nutrients in the HC differed: the Ca and P concentrations increased with reaction severity, while the S, K, Mg and Na concentrations in the HCs decreased or remained comparable to the dry faeces. Measurement of the nutrients in the ash after HC combustion showed a 10-fold increase in concentrations for almost all elements except for sulfur. During the HTC treatment, a considerable amount of nutrients was released to the liquid phase (PW). While the concentration of most nutrients in PW was similar for all test conditions, the NH _4_-N concentration showed a trend that higher reaction severity resulted in higher NH _4_-N values.

Table 4 summarizes the partitioning and fate of P and Mg through the hydrothermal treatment and subsequent HC combustion. These are of interest because they can be recovered further through struvite precipitation. Despite some differences in the concentration between test conditions ( Table 5), the total mass of each nutrient contained in HC was comparable for all tests. The increase in P concentration with higher reaction severity was compensated by the higher mass loss and lower HC yields, so that the mass of P in HC remained the same. Of the total mass in the feedstock, 75.6 – 81.0% P remained in HC, while 13.8 – 18.1% was found in PW. In contrast, Mg showed a relatively large partitioning ratio into the PW, ranging from 34.6 to 42.0%, while that for HC ranged from 52.6 to 61.5%. After the HC was combusted, both P and Mg remained in the ash, with more than 90% of the total amount recovered. In summary, the partitioning of nutrients was not dependent on the reaction severity of the test conditions employed in this study. 

**Table 4.** Phosphorus and magnesium balance through hydrothermal carbonization and subsequent combustion of hydrochar. PW=process water; HTC=hydrothermal carbonization.

Test ID	Total amount in HC (mg)	Partitioning ratio in HC (%)	Total amount in PW (mg)	Partitioning ratio in PW (%)	Loss from experimental procedure (%)	Total amount in Ash (mg)	Percentage from HC found in Ash (%)	Loss from experimental procedure + Loss from Combustion (%)
Phosphorus								
160-10	1084.9	79.0	205.3	15.0	6.0	1080.3	99.6	6.4
160-60	1089.9	79.4	197.4	14.4	6.2	1079.0	99.0	7.0
180-10	1069.6	77.9	211.7	15.4	6.7	1008.4	94.3	11.1
180-60	1038.2	75.6	232.9	17.0	7.4	1027.8	99.0	8.2
200-0	1048.0	76.3	230.9	16.8	6.9	1027.0	98.0	8.4
200-10	1077.5	78.5	248.3	18.1	3.4	1006.4	93.4	8.6
200-60	1112.2	81.0	189.9	13.8	5.2	1032.5	92.8	11.0
Faeces-1	feedstock 1373.2	Not applicable						
Magnesium								
160-10	345.2	61.5	194.1	34.6	3.9	342.3	99.2	4.5
160-60	335.1	59.7	195.1	34.7	5.6	322.0	96.1	7.9
180-10	312.9	55.7	206.6	36.8	7.5	281.5	90.0	13.1
180-60	295.2	52.6	224.7	40.0	7.4	285.3	96.6	9.2
200-0	304.0	54.2	224.8	40.0	5.8	299.1	98.4	6.7
200-10	297.9	53.1	235.8	42.0	4.9	271.0	91.0	9.7
200-60	320.8	57.1	215.3	38.3	4.6	289.7	90.3	10.1
Faeces-1	feedstock 561.5	Not applicable						

-   Average values of duplicates

**Table 5.** Nutrient contents in the feedstock, hydrochar, process water and hydrochar-ash samples.

Sample	S	Ca	K	Mg	Na	P	NH _4_-N ^†^
HC	mg / g						
160-10	4.4 ± 0.1	24.7	5.2 ± 0.3	4.7	1.0	14.9 ± 0.2	not available
160-60	4.5 ±0.2	25.6	5.7 ± 0.1	4.7 ± 0.1	1.1	15.3 ± 0.2	
180-10	4.2 ±0.1	27.4	5.1 ± 0.2	4.6	1.0	15.8 ± 0.1	
180-60	4.4	27.4	5.3 ± 0.1	4.5	1.0	16.0	
200-0	4.5 ± 0.2	26.9	5.2 ± 0.1	4.5	1.0	15.6 ± 0.1	
200-10	4.4 ± 0.1	28.5	5.4 ± 0.1	4.6	1.1	16.6	
200-60	4.4 ± 0.1	29.1	5.9 ± 0.2	5.1	1.2	17.6 ± 0.1	
Faeces-1	4.9 ± 0.2	20.8 ± 0.4	10.7 ± 0.6	5.8	1.9 ± 0.1	14.3 ± 0.2	
Ash	mg / g						
160-10	13.7 ± 0.3	260.1 ± 1.7	52.0 ± 0.5	49.7 ± 0.7	12.4 ± 0.2	156.9 ± 2.1	
160-60	12.0 ± 0.2	255.1 ± 2.9	51.0 ± 0.3	46.7 ± 0.6	11.6 ± 0.1	159.1 ± 1.8	
180-10	12.1 ± 0.1	254.4 ± 2.2	45.3 ± 0.3	42.6 ± 0.4	9.8 ± 0.1	152.6 ± 1.3	
180-60	12.9	261.9 ± 2.8	47.4 ± 0.6	43.2 ± 0.1	10.6 ± 0.2	157.6 ± 0.7	
200-0	13.7 ± 0.1	252.0 ± 2.5	49.3 ± 0.8	43.6 ± 0.3	11.2 ± 0.2	149.8 ± 0.7	
200-10	14.4 ± 0.2	259.2 ± 1.9	45.4 ± 0.8	41.7 ± 0.6	10.5 ± 0.1	155.0 ± 1.7	
200-60	13.6 ± 0.2	245.5 ± 1.0	45.7 ± 0.6	42.3 ± 0.1	10.8 ± 0.1	150.6 ± 0.5	
PW	mg / L						
160-10	373.1 ± 12.4	73.6 ± 2.2	2148.5 ± 64.4	733.0 ± 25.9	449.0 ± 12.6	775.2 ± 29.7	899.0
160-60	388.5 ± 6.5	59.2 ± 1.4	2106.0 ± 40.7	760.4 ± 11.5	433.3 ± 11.2	769.4 ± 13.5	604.4
180-10	384.6 ± 11.6	93.8 ± 3.3	2081.4 ± 99.2	773.4 ± 21.1	435.8 ± 23.6	792.4 ± 26.9	1033.0
180-60	403.6 ± 2.8	124.4 ± 1.9	2200.8 ± 46.9	821.3 ± 8.1	447.3 ± 3.7	851.1 ± 10.6	1265.3
200-0	412.5 ± 3.9	112.4 ± 0.8	2243.9 ± 63.9	807.6 ± 2.7	444.1 ± 3.6	829.5 ± 4.3	1257.3
200-10	406.9 ± 8.1	191.0 ± 0.8	2262.2 ± 6.0	850.8 ± 10.4	457.7 ± 1.7	895.9 ± 11.6	1367.6
200-60	399.8 ± 2.4	79.3 ± 0.8	2216.5 ± 41.4	785.2 ± 6.7	456.3 ± 5.4	692.8 ± 8.1	1571.5

-   Average value ± standard deviations n=3, noted when it is same or greater than 0.1. ^†^ Average values of duplicates

### Struvite precipitation

**Figure 2.**
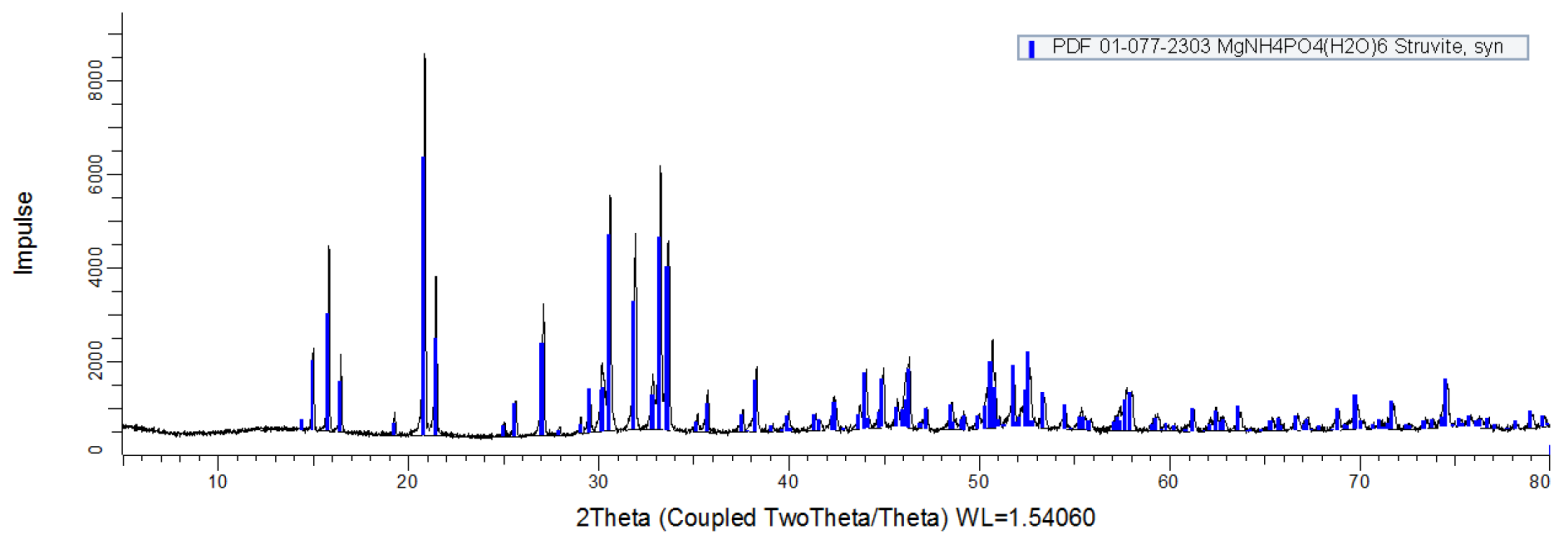
X-Ray diffraction analysis patterns from precipitate (Black), compared with the reference struvite (Blue).

The HC ash from Test-160-10, which yielded the best energy recovery ratio, was subjected to nutrient recovery experiments through struvite precipitation. The acid leaching method employed in this research showed high efficiencies of extraction. More than 95% of Mg and P in the ash were transferred to leachate. Results of precipitation experiments and nutrient balance are presented in Table 6. Approximately 3 / 4 of the nutrients in the solution was recovered as a form of a precipitate. The excess molar ratio of P compared to NH _4_-N and Mg suggests the possible formation of phosphate precipitate other than struvite to a limited extent in association with other cations, such as calcium phosphate. Also, this minor imbalance would be attributed to sensitivity differences in analytical techniques. Mg and P concentration of samples were measured by ICP-OES, while the N concentration was measured by CHN elemental analyser. The XRD patterns obtained from the precipitate matched well with the reference (PDF 01-077-2303, ICDD) from struvite ( Figure 2).

**Table 6.** Experimental results and nutrient balance for struvite precipitation. PW=process water.

		^†^Precipitation experiment			^‡^Extrapolated values for full utilization of ash (proportion in total amount, %)		
		NH _4_-N	Mg	P	NH _4_-N	Mg	P
Input	PW	1.66	0.78	0.65	17.0 (42.4)	8.0 (20.0)	6.6 (16.5)
	Ash leachate	-	0.42	1.01	-	14.1 (35.2)	33.5 (83.5)
	Mg supplement	-	0.46	-	-	18.0 (44.8)	-
	NH _4_ supplement	-	-	-	23.1 (57.6)	-	-
Total in solution		1.66	1.66	1.66	40.1	40.1	40.1
Output	Precipitate	* 1.24	1.29	1.31	30.0	31.2	31.6
	Precipitation efficiency (%)	74.7	77.7	78.9			

† Average values of duplicates, mmol ‡ Speculated values derived from the experimental results, mmol * Elemental nitrogen obtained from CHN analysis

If the HC-ash and PW from the selected batch of HTC are exclusively used for nutrient recovery, the molar ratio among available P: NH _4_-N: Mg would be 1: 0.42: 0.55 ( Table 4). It indicates that the corresponding supplements for NH _4_-N and Mg are necessary to establish a stoichiometric molar ratio of 1: 1: 1 for struvite recovery. When these elements are provided, around 30 mmol of struvite, corresponding to 7.4 g MgNH _4_PO _4_⋅6H _2_O, can be harvested from hydrothermal treatment of 96 g dry faeces. After separation of the precipitate, around 1 / 4 of the total nutrient input was available in the solution at pH 8. Expected usage of acid (1M H _2_SO _4_) and base (1M NaOH) in this case during the extraction-precipitation processes were 76.5 and 39.0 mL, respectively.

## Discussion

### Scenario for energy and nutrient recovery

This research presents a proof of concept for the hydrothermal treatment of faecal waste as an alternative sanitation technology in terms of technical feasibility, hygienisation and resource recovery of energy and nutrients. The low-cost HTC reactor with a relatively simple design and function performed well in comparison with a commercial reactor. The faecal waste was sanitised in all test conditions employed in this research, since they were greater than or comparable to the previously reported HTC conditions (at 150 °C for 30 min) for complete elimination of pathogens and microbial DNA ( Ducey *et al*., 2017). For all test conditions employed in the optimization experiments, more than 80% of the initial energy content in the feedstock was recaptured in the resulting materials. Phosphorus recovery was also demonstrated to be possible. Overall, 72% of the phosphorus in the faeces could be recovered as a solid struvite, which can be used as a slow-releasing fertiliser. Further research will be necessary to determine the overall nutrient recovery throughout the process. 

**Figure 3.**
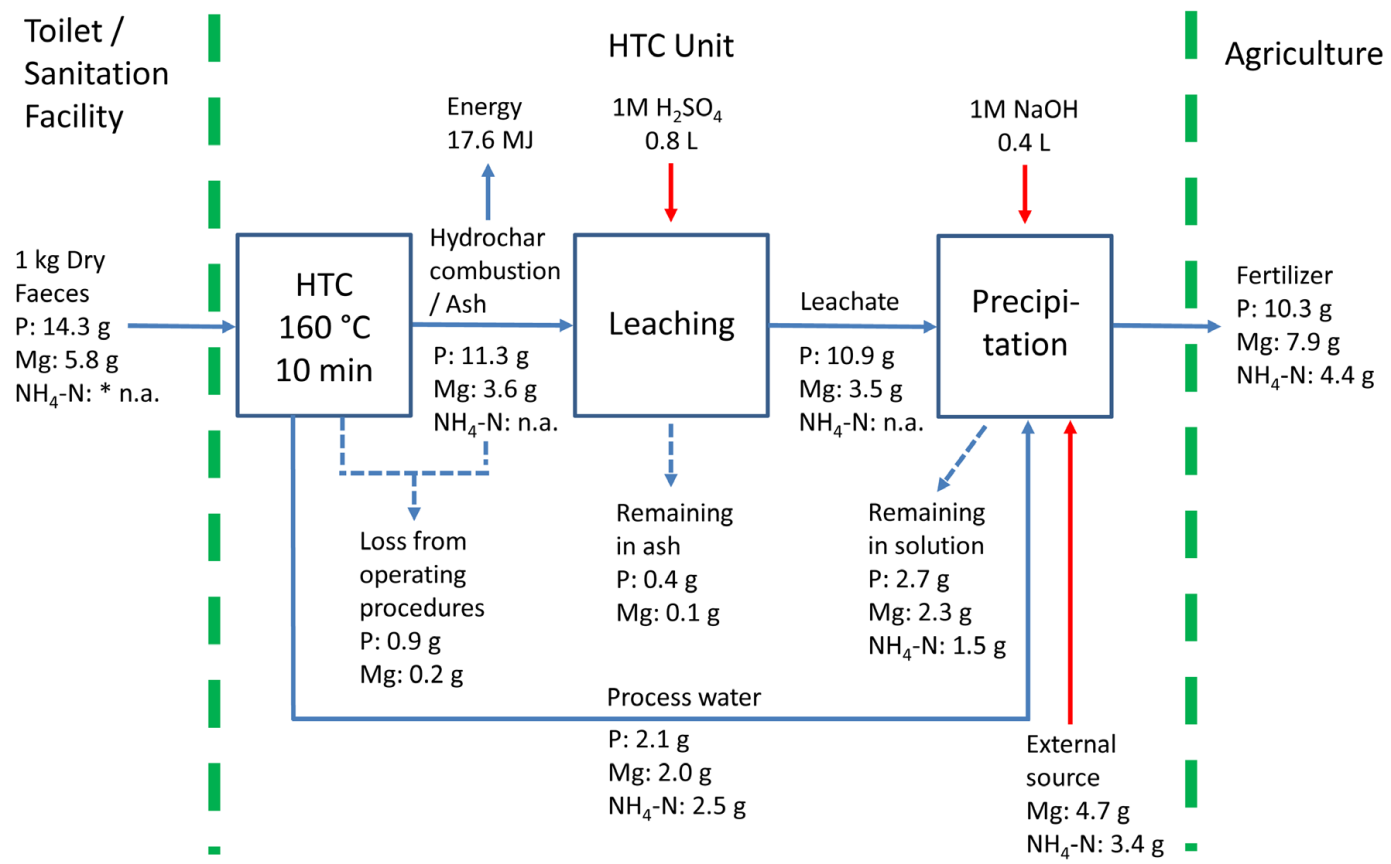
Scenario for energy and nutrient recovery through hydrothermal treatment of faecal waste (Note: The material balance for P and Mg is complete, but only partial results are given for NH _4_-N. *n.a. not available). HTC=hydrothermal carbonization.

Therefore, based on the tests carried out in this research, we propose a process combination for energy and nutrient recovery through hydrothermal treatment of faecal waste. The best-case scenario for resource recovery is depicted in Figure 3. In this scenario, hydrothermal treatment of 1kg dry faeces (at 160 °C for 10 min) can produce 17.6 MJ and 10.3 g phosphorus in the form of solid fuel and struvite, respectively. Since the experimental results showed similar values for total energy recovery at all test conditions, the HTC condition with the lowest severity was used as the basis for the scenario. It is a more appealing option in terms of less energy consumption and ease of relevant reactor development. Furthermore, due to the limited energy potential of anaerobic digestion (AD) of PW (0.9 MJ. <6% of energy invested), we assumed that PW would be exclusively used in nutrient recovery. 

The calorific values of feedstock and resulting HCs in this research correspond well with previous investigations on hydrothermal treatment of fresh human faeces ( Afolabi *et al*., 2017; Yahav Spitzer *et al*., 2018). The HHVs of HCs ranged from 23.2 to 25.2 MJ/kg, which lies in the range of low bituminous coal, charcoal briquette and pellets produced by dry pyrolysis of human faeces. These values are greater than many of the biomass fuels currently used in the developing world for domestic uses such as cooking and heating ( Ward *et al*., 2014). For industrial applications, co-combustion with coal can be a considerable option ( Chen *et al.*, 2020). In addition to high HHVs, the low sulfur (~0.5%) and ash (~10%) contents can be advantages as a clean energy source. In contrast, it has to be noted that relatively high nitrogen content of HCs (3 - 4%, dry weight basis) would result in more nitrogen oxides (NOx) compounds formation in the exhaust gas (cf. coals typically have nitrogen contents below 2%)( Baxter *et al.*, 1996). Further combustion analysis on HCs is necessary to assure suitability for solid fuel application.

AD has been considered a promising complement to HTC for PW treatment in terms of energy recovery and degradation of organic contaminants ( Lucian *et al.*, 2020; Wirth & Mumme, 2013). As far as we know, the present study is the first experimental investigation on AD of PW derived from raw human waste. The BMP values of PWs obtained in this work ranged from 0.16 to 0.24 L CH4 / g VS (corresponding to 0.33 - 0.46 MJ / L PW). Previous studies on untreated human waste reported slightly higher BMPs ranging from 0.26 to 0.36 L CH4 / g VS ( Kim *et al.*, 2019; Rajagopal *et al.*, 2013; Riungu *et al.*, 2019). Though it is apparent that the hydrothermal process can increase the anaerobic biodegradability of various biomasses mainly through solubilisation of organic substances in the liquid phase and hydrolytic breakdown of rigid substances, applying high HTC temperature over 200 °C may hamper the AD process because of the formation and accumulation of inhibitors such as volatile fatty acids ( Gaur *et al.*, 2020; Parmar & Ross, 2019). Investigations on the co-anaerobic digestion of PW and HC would induce beneficial effects of HC supplements in several pathways: i) facilitating electron transfer (DIET: direct interspecies electron transfer) in the microbiome, ii) reducing inhibitory effects from high ammonium concentration, iii) decreasing the lag phase, iv) supplying nutrients, v) increasing alkalinity and production of intermediates, e.g. acetic acid and hydrogen and vi) retaining a part of CO2 generated during the AD process ( Ipiales *et al.*, 2021).

Throughout the processes, the phosphorus contained in the faeces was well retained in the system boundary. In total, 90% of the initial phosphorus in the feedstock was delivered to the precipitation unit, and finally, 72% can be recovered in a solid phase. Because a considerable amount of nutrients were still present in the solution after separation of precipitate, further research for its reuse or enhancing the precipitation efficiency is recommended. The provision of surplus Mg over the stoichiometric ratio would be an option for better P recovery ( Rahaman *et al*., 2008). pH adjustments should also be investigated. While an increase in pH would improve the precipitation efficiency ( Nelson *et al*., 2003), an overdose of base over pH 10.5 can facilitate the formation of magnesium hydroxide compounds rather than struvite ( Kim *et al*., 2017).

Even though the production of struvite is an appealing option that can be marketed as a slow-releasing fertiliser, it may not be economical due to the cost burden from the post-processes such as acid leaching and precipitation. A more straightforward nutrient recovery option can be the direct application of resulting products from HTC. Because the HTC products may contain various chemical substances (e.g. organic acids and furan compounds) which can be beneficial or detrimental to plant growth, phytotoxicity tests with relevant conditions employing target crop and soil type need to be performed prior to their implementation in agriculture. It was reported that the increase in reaction severity resulted in greater phytotoxicity ( Bargmann *et al*., 2013). The phytotoxic compounds found in the HC in previous research were reported to be water-soluble and volatile ( Bargmann *et al*., 2013; Celletti *et al*., 2021). Therefore, washing and/or ageing of HC can be appropriate post-treatments for decreasing the adverse impacts.

Some aspects still need further investigation, such as the dewaterability of HC which can be important in the practical implementation of HTC technology by causing additional costs for separation of the solid and liquid phase of the resulting slurry. Studies have found that higher reaction severity increases the hydrophobicity of HC, which is associated with dewaterability ( Sharma & Dubey, 2020; Zhang *et al*., 2019). Though it was not quantitatively investigated in this study, observation during the experiments agrees with these reports. While the Test-160-10 with the lowest SF showed the best energy recovery ratio, its HC had the lowest filterability. Because we used filters with a relatively small pore size (11 µm), employing filters with a greater pore size (e.g. 63 µm) ( Afolabi & Sohail, 2017) would be one of the simplest options to be tested in future investigations.

Energy use for heating the reactor can be a major operational cost factor in such a treatment system. The HCs obtained from Test-160-10 and BLR-160-10 had greater energy values than what was invested for each treatment. However, the results must be interpreted carefully and not be considered as an indication for suggesting energy independence (no external energy input needed) of treatment concept, because i) the energy input in the commercial reactor system only measured the electric energy used for heating the vessel; energy input from other units such as process parameter monitoring and controlling devices might be significant, and ii) the HHV of HC was obtained by bomb calorimetry carried out under surplus provision of oxygen. The combustion efficiency in practical use may not correspond with the laboratory results.

Also, on the laboratory scale, the energy efficiency of the system varies depending on the scale of the equipment. Water tests performed with a batch reactor system using different capacities of pressure vessels showed a significant variance in energy efficiency. The greater volume of the reactor resulted in better energy efficiency (data not shown). For a more reliable estimate of the energy demand for a real scale implementation, further research on a pilot-scale low-cost reactor system with direct energy consumption measurement is required to establish a better understanding and a model for the energy balance.

### Technical feasibility of low-cost reactor concept

We developed a low-end reactor using widely available materials and operated it with a non-electric heating method. To our knowledge, this is the first attempt to use direct combustion of fuel for heating the reactor. Previous research mostly employed electric devices such as a heating mantle, microwave, oil bath or oven. This study demonstrates the feasibility of such a system and the results hold promise for the further development of small scale, decentralised and/or mobile implementations of HTC, with a possibility of being fuelled by combustion of biowastes such as crop residues or dry faecal sludge. In the current design, the amount of instrumentation was minimized in order to keep the costs down, but ensure safe operation. Of particular interest in terms of the safety assurance of low-cost reactor systems is the monitoring of the pressure increase over the heating time. Two phenomena can affect the pressure and must be taken into account in the further reactor design and operation: i) when a closed vessel containing water and feedstock with a certain headspace is heated, pressure increases due to the saturated water vapour and gas generation from feedstock, ii) the region where the thermally expanded water fills the vessel completely, the subcooled liquid compression region, must be avoided. Because the pressure increases very vigorously in this region with a slight increase in temperature, the relevant experimental parameters (e.g. reactor volume, temperature, water volume and dry matter loading) should be designed carefully ( Ro *et al*., 2020).

Our observations from Test-200-0, 200-10, 200-60 and BLR-200-0 show evidence that the gas generation from feedstock can have a significant effect on the pressure increase, when it is exposed to high temperatures for a prolonged duration ( Table 1 and Figure 1). Given the specifications of the current BLR design, HTC conditions more severe than BLR-200-0 would be inappropriate because of the rapid increase in pressure seen around 200 °C. It is reassuring, however, that for all test conditions the pressure inside the reactor immediately started to decrease when the heating was stopped. It was likely that the exothermic energy from the feedstock was not sufficient for continuing the reaction ( Reza *et al*., 2014) which can induce further pressure development and cause pressure risk. Also, a recent study reported that the exothermicity of HTC has only a minor impact on the overall energy balance, especially when compared to the energy required for heating feedstock ( Ischia *et al.*, 2022). 

Since most of the energy input to the reactor was used for heating the reactor and water ( Table 3), it may be beneficial to increase the DM loading to maximize treatment capacity and energy recovery. An important topic for future research is to investigate the interactions between the DM loading and its effects on pressure development and the properties of the resulting product.

### Positioning HTC in current sanitation technologies

The HTC process can be coupled with many existing sanitation technologies due to its ability to hygienise faecal matter and to increase its fuel value. However, the specific properties of HC and PW are largely determined by the feedstock characteristics ( Libra *et al*., 2011), which can vary considerably. The characteristics of faecal sludge generated from onsite treatment facilities depends on several factors such as treatment technology, storage duration, infiltration / leakage (from the environment to system, or vice versa), collection method and climate ( Strande *et al*., 2014). In this context, it is noteworthy that the feedstock used in this research was prepared by autoclaving, drying at 105°C, grinding and remoisturizing to assure the microbial safety and reproducibility in the laboratory experiments. These pre-treatments would have altered properties of raw faeces (e.g. loss of volatile substances such as ammonium and change in dewaterability) to a certain extent which might affect the characteristics of resulting materials.

The sanitation technology that best matches the approach used in this study would be the Urine-Diverting-Dry-Toilet (UDDT) which results in undiluted raw faeces that need further treatment ( Riungu *et al*., 2019). Another possible application for HTC would be as a post-treatment unit of sludge drying bed facilities, where the final product is of insufficient quality with a relatively low heating value (12 MJ/kg) and microbial risks ( Seck *et al*., 2015). Integration of hydrothermal treatment in the system can mitigate the limitations of the final product in terms of fuel quality and microbial safety assurance. The use of the BLR which could be heated by direct biomass combustion for the HTC treatment is an attractive option. The treatment could be fuelled by combusting raw dry sludge as a low-cost fuel. In the case of faecal sludge with high moisture contents, co-HTC with other dry biowastes may be a more appealing option. Since biomass usually have a lower specific heat capacity (1.3 – 1.5 J / g dry weight at 40 °C) than water ( Dupont *et al*., 2014), its supplements will decrease the energy input for a treatment batch while producing more HC products. Also, a greater heat of reaction will be obtained from having more organic substrate in the treatment batch. A previous study reported that co-HTC of swine manure and lignocellulosic biomass showed a meaningful increase in HHV and energy content of the resulting HC materials compared to the one obtained from the treatment of pure swine manure. In addition, the concentration and bioavailability of heavy metals in HC decreased by the co-HTC ( Lang *et al*., 2018).

## Conclusions

The current rate of progress in sanitation service provision in LDCs calls for breakthroughs. HTC can serve as an attractive technical solution that eliminates the microbial risk of faecal waste and converts it into valuable resources. This research provides a quantitative evaluation of the hydrothermal treatment of human waste and resource recovery. We verified the low-cost reactor concept by constructing the BLR and carrying out successive tests for faecal waste treatment. The BLR was constructed using widely available materials and technology so that local production of such hydrothermal reactors in target regions can be implemented. Also, the stable operation of the reactor using a non-electric heating method allows a wide range of heating sources to be selected. The results show that low reaction severity treatment is recommended to reduce energy consumption for treatment and increase ease of reactor construction. The resulting HC materials showed competitive heating values as solid fuel. Struvite production from HC ash and PW was demonstrated to be an efficient phosphorus recovery pathway.

Interesting future research topics include:

- phytotoxicity and plant-available nutrient analyses for faeces-derived HC, HC-ash and PW materials
- optimization of dry matter loading in HTC for enhancing energy recovery
- hydrothermal treatment of various faecal sludge samples in field conditions
- co-treatment of faecal sludge and biowaste (e.g. agricultural residue)
- pilot-scale low-cost reactor development based on locally available resources in target countries

## Data availability

### Underlying data

Zenodo: Underlying data / Hydrothermal carbonization as an alternative sanitation technology: process optimization and development of low-cost reactor. https://doi.org/10.5281/zenodo.6375576 ( Chung *et al*., 2021).

This project contains the following underlying data:

- HTC as an alternative sanitation technology_underlying data_R1.xlsx
- Selected HTC pressure-temperature profile.xlsx
- XRD Struvite.zip

### Extended data

Zenodo: Design and technical drawing of a bench-scale low-cost hydrothermal carbonization reactor. https://doi.org/10.5281/zenodo.5094908 ( Chung, 2021).

This project contains the following extended data:

- 1. Design consideration.pdf
- 2. Design calculation.pdf
- 3. Technical drawing.pdf
- 4. Material list.xls
- 5. 3D visualization.stp

Data are available under the terms of the Creative Commons Attribution 4.0 International license (CC-BY 4.0).

## References

[ref-1] Afolabi OOD Sohail M : Comparative evaluation of conventional and microwave hydrothermal carbonization of human biowaste for value recovery. *Water Sci Technol.* 2017;75(12):2852–2863. 10.2166/wst.2017.164 28659525

[ref-2] Afolabi OOD Sohail M Thomas CLP : Characterization of Solid Fuel Chars recovered from Microwave Hydrothermal Carbonization of Human Biowaste. *Energy (Oxf).* 2017;134:74–89. 10.1016/j.energy.2017.06.010 33343060 PMC7680956

[ref-3] Bargmann I Rillig MC Buss W : Hydrochar and Biochar Effects on Germination of Spring Barley. *J Agron Crop Sci.* 2013;199(5):360–373. 10.1111/jac.12024

[ref-70] Baxter LL Mitchell RE Fletcher TH : Nitrogen Release during Coal Combustion. *Energy Fuels.* 1996;10(1):188–196. 10.1021/ef9500797

[ref-4] Bleuler M Gold M Strande L : Pyrolysis of Dry Toilet Substrate as a Means of Nutrient Recycling in Agricultural Systems: Potential Risks and Benefits. *Waste Biomass Valor.* 2021;12(7):4171–4183. 10.1007/s12649-020-01220-0

[ref-5] Boot NL Scott RE : Faecal sludge in Accra, Ghana: problems of urban provision. *Water Sci Technol.* 2009;60(3):623–631. 10.2166/wst.2009.441 19657157

[ref-6] Celletti S Bergamo A Benedetti V : Phytotoxicity of hydrochars obtained by hydrothermal carbonization of manure-based digestate. *J Environ Manage.* 2021;280:111635. 10.1016/j.jenvman.2020.111635 33187784

[ref-100] Chen L Wen C Wang W : Combustion behaviour of biochars thermally pretreated via torrefaction, slow pyrolysis, or hydrothermal carbonisation and co-fired with pulverised coal. *Renewable Energy.* 2020;161:867–877. 10.1016/j.renene.2020.06.148

[ref-8] Chung JW : Design and technical drawing of a bench-scale low-cost hydrothermal carbonization reactor. *Zenodo.* 2021. 10.5281/zenodo.5094908

[ref-7] Chung JW Ovsyannikova E Treichler A : Underlying data / Hydrothermal carbonization as an alternative sanitation technology: process optimization and development of low-cost reactor. *Zenodo.* 2021. 10.5281/zenodo.6375576 PMC10446067 37645161

[ref-9] Cordell D Drangert JO White S : The story of phosphorus: Global food security and food for thought. *Glob Environ Change.* 2009;19(2):292–305. 10.1016/j.gloenvcha.2008.10.009

[ref-10] Cordell D Rosemarin A Schröder JJ : Towards global phosphorus security: A systems framework for phosphorus recovery and reuse options. *Chemosphere.* 2011;84(6):747–758. 10.1016/j.chemosphere.2011.02.032 21414650

[ref-11] Danso-Boateng E Holdich RG Martin SJ : Process energetics for the hydrothermal carbonisation of human faecal wastes. *Energy Convers Manag.* 2015;105:1115–1124. 10.1016/j.enconman.2015.08.064

[ref-12] Danso-Boateng E Holdich RG Shama G : Kinetics of faecal biomass hydrothermal carbonisation for hydrochar production. *Appl Energy.* 2013;111:351–357. 10.1016/j.apenergy.2013.04.090

[ref-13] Desmidt E Ghyselbrecht K Zhang Y : Global Phosphorus Scarcity and Full-Scale P-Recovery Techniques: A Review. *Crit Rev Environ Sci Technol.* 2015;45(4):336–384. 10.1080/10643389.2013.866531

[ref-14] Ducey TF Collins JC Ro KS : Hydrothermal carbonization of livestock mortality for the reduction of pathogens and microbially-derived DNA. *Front Environ Sci Eng.* 2017;11(3):9. 10.1007/s11783-017-0930-x

[ref-15] Dupont C Chiriac R Gauthier G : Heat capacity measurements of various biomass types and pyrolysis residues. *Fuel.* 2014;115:644–651. 10.1016/j.fuel.2013.07.086

[ref-16] Fakkaew K Koottatep T Polprasert C : Faecal sludge treatment and utilization by hydrothermal carbonization. *J Environ Manage.* 2018;216:421–426. 10.1016/j.jenvman.2017.09.031 28941833

[ref-51] Gaur RZ Khoury O Zohar M : Hydrothermal carbonization of sewage sludge coupled with anaerobic digestion: Integrated approach for sludge management and energy recycling. *Energy Convers Manag.* 2020;224:113353. 10.1016/j.enconman.2020.113353

[ref-50] Heidari M Norouzi O Salaudeen S : Prediction of Hydrothermal Carbonization with Respect to the Biomass Components and Severity Factor. *Energy Fuels.* 2019;33(10):9916–9924. 10.1021/acs.energyfuels.9b02291

[ref-52] Ipiales RP de la Rubia MA Diaz E : Integration of Hydrothermal Carbonization and Anaerobic Digestion for Energy Recovery of Biomass Waste: An Overview. *Energy Fuels.* 2021;35(21):17032–17050. 10.1021/acs.energyfuels.1c01681

[ref-53] Ischia G Cazzanelli M Fiori L : Exothermicity of hydrothermal carbonization: Determination of heat profile and enthalpy of reaction via high-pressure differential scanning calorimetry. *Fuel.* 2022;310:122312. 10.1016/j.fuel.2021.122312

[ref-17] Kim D Min KJ Lee K : Effects of pH, molar ratios and pre-treatment on phosphorus recovery through struvite crystallization from effluent of anaerobically digested swine wastewater. *Environ Eng Res.* 2017;22(1):12–18. Reference Source

[ref-54] Kim J Kim J Lee C : Anaerobic co-digestion of food waste, human feces, and toilet paper: Methane potential and synergistic effect. *Fuel.* 2019;248:189–195. 10.1016/j.fuel.2019.03.081

[ref-55] Konrad O Hasan C Marder M : Comparison of two gas volume measurement systems by evaluating biochemical methane potential. *Environ Qual Manag.* 2021;31(3):201–207. 10.1002/tqem.21776

[ref-18] Lang Q Guo Y Zheng Q : Co-hydrothermal carbonization of lignocellulosic biomass and swine manure: Hydrochar properties and heavy metal transformation behavior. *Bioresour Technol.* 2018;266:242–248. 10.1016/j.biortech.2018.06.084 29982044

[ref-19] Libra JA Ro KS Kammann C : Hydrothermal carbonization of biomass residuals: a comparative review of the chemistry, processes and applications of wet and dry pyrolysis. *Biofuels.* 2011;2(1):71–106. 10.4155/bfs.10.81

[ref-56] Lucian M Volpe M Merzari F : Hydrothermal carbonization coupled with anaerobic digestion for the valorization of the organic fraction of municipal solid waste. *Bioresour Technol.* 2020;314:123734. 10.1016/j.biortech.2020.123734 32622280

[ref-20] Mallory A Holm R Parker A : A Review of the Financial Value of Faecal Sludge Reuse in Low-Income Countries. *Sustainability.* 2020;12(20):8334. 10.3390/su12208334

[ref-21] Namioka T Miyazaki M Morohashi Y : Modeling and Analysis of Batch-Type Thermal Sludge Pretreatment for Optimal Design. *Journal of Environment and Engineering.* 2008;3(1):170–181. 10.1299/jee.3.170

[ref-22] Nelson NO Mikkelsen RL Hesterberg DL : Struvite precipitation in anaerobic swine lagoon liquid: effect of pH and Mg:P ratio and determination of rate constant. *Bioresour Technol.* 2003;89(3):229–236. 10.1016/s0960-8524(03)00076-2 12798112

[ref-23] Ovsyannikova E Kruse A Becker GC : Feedstock-Dependent Phosphate Recovery in a Pilot-Scale Hydrothermal Liquefaction Bio-Crude Production. *Energies.* 2020;13(2):379. 10.3390/en13020379

[ref-24] Ovsyannikova E Kruse A Becker GC : Valorization of Byproducts from Hydrothermal Liquefaction of Sewage Sludge and Manure: the Development of a Struvite-Producing Unit for Nutrient Recovery. *Energy & Fuels.* 2021;35(11):9408–9423. 10.1021/acs.energyfuels.1c00561

[ref-57] Parmar KR Ross AB : Integration of Hydrothermal Carbonisation with Anaerobic Digestion; Opportunities for Valorisation of Digestate. *Energies.* 2019;12(9):1586. 10.3390/en12091586

[ref-25] Petzet S Peplinski B Cornel P : On wet chemical phosphorus recovery from sewage sludge ash by acidic or alkaline leaching and an optimized combination of both. *Water Res.* 2012;46(12):3769–3780. 10.1016/j.watres.2012.03.068 22579406

[ref-26] Rahaman MS Ellis N Mavinic DS : Effects of various process parameters on struvite precipitation kinetics and subsequent determination of rate constants. *Water Sci Technol.* 2008;57(5):647–654. 10.2166/wst.2008.022 18401133

[ref-58] Rajagopal R Lim JW Mao Y : Anaerobic co-digestion of source segregated brown water (feces-without-urine) and food waste: for Singapore context. *Sci Total Environ.* 2013;443:877–886. 10.1016/j.scitotenv.2012.11.016 23247290

[ref-101] Reza MT Andert J Wirth B : Hydrothermal Carbonization of Biomass for Energy and Crop Production. *Applied Bioenergy.* 2014;1:11–29. Reference Source

[ref-27] Reza MT Andert J Wirth B : Hydrothermal Carbonization of Biomass for Energy and Crop Production. *Applied Bioenergy.* 2014;1(1):11–29. 10.2478/apbi-2014-0001

[ref-28] Riungu J Ronteltap M van Lier JB : Anaerobic stabilisation of urine diverting dehydrating toilet faeces (UDDT-F) in urban poor settlements: biochemical energy recovery. *J Water Sanit Hyg Dev.* 2019;9(2):289–299. 10.2166/washdev.2019.099

[ref-29] Ro KS Libra JA Alvarez-Murillo A : Comparative Studies on Water- and Vapor-Based Hydrothermal Carbonization: Process Analysis. *Energies.* 2020;13(21):5733. 10.3390/en13215733

[ref-30] Robbiani Z : Hydrothermal carbonization of biowaste/fecal sludge. Master's thesis, Swiss Federal Institute of Technology Zurich,2013. Reference Source

[ref-31] Seck A Gold M Niang S : Faecal sludge drying beds: increasing drying rates for fuel resource recovery in Sub-Saharan Africa. *J Water Sanit Hyg Dev.* 2015;5(1):72–80. 10.2166/washdev.2014.213

[ref-32] Sharma HB Dubey BK : Binderless fuel pellets from hydrothermal carbonization of municipal yard waste: Effect of severity factor on the hydrochar pellets properties. *J Clean Prod.* 2020;277:124295. 10.1016/j.jclepro.2020.124295

[ref-33] Sharma R Jasrotia K Singh N : A Comprehensive Review on Hydrothermal Carbonization of Biomass and its Applications. *Chemistry Africa.* 2020;3(1):1–19. 10.1007/s42250-019-00098-3

[ref-34] Song C Zheng H Shan S : Low-Temperature Hydrothermal Carbonization of Fresh Pig Manure: Effects of Temperature on Characteristics of Hydrochars. *J Environ Engineer.* 2019;145(6):04019029. 10.1061/(ASCE)EE.1943-7870.0001475

[ref-35] Strande L Ronteltap M Brdjanovic D : Faecal Sludge Management: Systems Approach for Implementation and Operation.IWA Publishing,2014;13. 10.2166/9781780404738

[ref-36] VDI: Fermentation of organic materials - Characterisation of the substrate, sampling, collection of material data, fermentation tests.Verein Deutscher Ingenieure Düsseldorf, Germany,2006. Reference Source

[ref-59] Velkushanova K Strande L Ronteltap M : Methods for Faecal Sludge Analysis. IWA Publishing.2021. Reference Source

[ref-37] Ward BJ Yacob TW Montoya LD : Evaluation of Solid Fuel Char Briquettes from Human Waste. *Environ Sci Technol.* 2014;48(16):9852–9858. 10.1021/es500197h 25020243

[ref-38] WHO, UNICEF: Progress on household drinking water, sanitation and hygiene 2000-2020: five years into the SDGs.Geneva: World Health Organization (WHO) and the United Nations Children’s Fund (UNICEF), Licence: CC BY-NC-SA 3.0 IGO,2021. Reference Source

[ref-60] Wirth B Mumme J : Anaerobic Digestion of Waste Water from Hydrothermal Carbonization of Corn Silage. *Applied Bioenergy.* 2013;1(1). 10.2478/apbi-2013-0001

[ref-39] Wüst D Rodriguez Correa C Suwelack KU : Hydrothermal carbonization of dry toilet residues as an added-value strategy - Investigation of process parameters. *J Environ Manage.* 2019;234:537–545. 10.1016/j.jenvman.2019.01.005 30660054

[ref-40] Yahav Spitzer R Mau V Gross A : Using hydrothermal carbonization for sustainable treatment and reuse of human excreta. *J Clean Prod.* 2018;205:955–963. 10.1016/j.jclepro.2018.09.126

[ref-41] Zhang S Zhu X Zhou S : Biochar from Biomass and Waste.Ok, Y.S., Tsang, D.C.W., Bolan, N. and Novak, J.M. (eds), Elsevier,2019;275–294. 10.1016/C2016-0-01974-5

