# Supplementary material for: Hydrothermal carbonization as an alternative sanitation technology: process optimization and development of low-cost reactor
Source: Open Res Eur. 2022 Jun 10;1:139. Originally published 2021 Nov 26. [Version 2] doi: 10.12688/openreseurope.14306.2 (PMC10446067; doi:10.12688/openreseurope.14306.2)
Supplement: Peer review history

# Peer review: Reviewer response for version 2

**DOI:** 10.21956/openreseurope.15792.r29511

**Published:** 2022-08-22

**Authors:** Amit Gross

**Affiliations:** 1 Zuckerberg Institute for Water Research, The Jacob Blaustein Institutes for Desert Research, Ben-Gurion University of the Negev, Be'er Sheva, Israel

The authors have addressed all comments and therefore I recommend it for indexing as is.

Is the study design appropriate and does the work have academic merit?

Partly

Is the work clearly and accurately presented and does it cite the current literature?

Yes

If applicable, is the statistical analysis and its interpretation appropriate?

Partly

Are all the source data underlying the results available to ensure full reproducibility?

Yes

Are the conclusions drawn adequately supported by the results?

Partly

Are sufficient details of methods and analysis provided to allow replication by others?

Yes

Reviewer Expertise:

NA

I confirm that I have read this submission and believe that I have an appropriate level of expertise to confirm that it is of an acceptable scientific standard.

# Peer review: Reviewer response for version 2

**DOI:** 10.21956/openreseurope.15792.r29512

**Published:** 2022-06-15

**Authors:** Luca Fiori, Donato Scrinzi

**Affiliations:** 1 Department of Civil, Environmental and Mechanical Engineering & C3A - Center Agriculture Food Environment, University of Trento, Trento, Italy; 2 Department of Civil, Environmental and Mechanical Engineering, University of Trento, Trento, Italy

The authors addressed all our requests for improvement and clarification that we made in revising the original manuscript.

 The quality of the revised manuscript is such that it is now acceptable for indexing.

Is the study design appropriate and does the work have academic merit?

Yes

Is the work clearly and accurately presented and does it cite the current literature?

Partly

If applicable, is the statistical analysis and its interpretation appropriate?

I cannot comment. A qualified statistician is required.

Are all the source data underlying the results available to ensure full reproducibility?

Yes

Are the conclusions drawn adequately supported by the results?

Yes

Are sufficient details of methods and analysis provided to allow replication by others?

Partly

Reviewer Expertise:

We (Luca Fiori and Donato Scrinzi) feel qualified to review and assess all the aspects of the paper.

We confirm that we have read this submission and believe that we have an appropriate level of expertise to confirm that it is of an acceptable scientific standard.

# Peer review: Reviewer response for version 1

**DOI:** 10.21956/openreseurope.15435.r28090

**Published:** 2022-01-18

**Authors:** Amit Gross

**Affiliations:** 1 Zuckerberg Institute for Water Research, The Jacob Blaustein Institutes for Desert Research, Ben-Gurion University of the Negev, Be'er Sheva, Israel

I appreciate your work, I have enjoyed reading it. Overall I think it is a good paper with scientific merit to HTC of human feces. I do have some fairly minor comments and questions.

Method section:

- I could not understand the decision to sort different components because it does not represent correctly human excretions nor the overall waste from a composting toilet. Thus, not really represent any real feedstock. Can you comment on the reasoning?
- Statistical analysis is somewhat weak, specifically as you used only duplicates.

Results Section:

- For clarity please revise Figure 1 legends.

Discussion section

- Please add a discussion about the quality of the char for combustion.

Is the study design appropriate and does the work have academic merit?

Partly

Is the work clearly and accurately presented and does it cite the current literature?

Yes

If applicable, is the statistical analysis and its interpretation appropriate?

Partly

Are all the source data underlying the results available to ensure full reproducibility?

Yes

Are the conclusions drawn adequately supported by the results?

Partly

Are sufficient details of methods and analysis provided to allow replication by others?

Yes

Reviewer Expertise:

Currently study HTC of organic waste including human excretion.

I confirm that I have read this submission and believe that I have an appropriate level of expertise to confirm that it is of an acceptable scientific standard, however I have significant reservations, as outlined above.

# Response

**Published:** 2022-05-24

**Authors:** Jae Wook Chung

**Affiliations:** Zurich University of Applied Sciences, Switzerland

Dear Reviewers, We appreciate your comments. They were beneficial in improving this article, making it more precise and comprehensive. We accepted most of your comments and revised the manuscript accordingly. Please find our responses to the comments for the cases we would like to maintain the original manuscript or need further clarification. We hope the second version of the manuscript addresses your comments sufficiently.  

***Reviewer 2 / comments*** *I appreciate your work, I have enjoyed reading it. Overall I think it is a good paper with scientific merit to HTC of human feces. I do have some fairly minor comments and questions.*

*Method section:*

- *I could not understand the decision to sort different components because it does not represent correctly human excretions nor the overall waste from a composting toilet. Thus, not really represent any real feedstock. Can you comment on the reasoning?*

**Our response:** We agree that the faecal stock used in the HTC experiments does not represent any raw faecal sludges in field conditions. Pre-treatments of faeces were unavoidable to meet the biosafety requirement in laboratory research. This aspect is described in the Methods and Discussion section of the revised manuscript as recommended by Reviewer 1. As described in the discussion section, the property of faecal sludge varies greatly depending on many factors. Thus, we selected pure human waste as the main component of various faecal sludges, especially ones obtained from Urine Diverting Dry Toilet or box sanitation. As an alternative to sorting out faecal mass, we tested autoclaving-drying-grinding of faecal stocks as received (faeces + toilet paper + woody cover materials). However, it was challenging to prepare homogeneous feedstock because the high fibre content of large paper materials interrupted the mechanical grinding process. The most reliable and necessary investigation would be testing the field samples in LDC conditions. It is suggested as further research topics.  

- *Statistical analysis is somewhat weak, specifically as you used only duplicates.*

**Our response:** Performing at least triplicate of each experimental condition would have provided a more robust statistical basis. However, one of the main difficulties of this research was collecting enough undiluted human faeces. Based on the minor differences observed from the duplicate of each experimental condition (Table 1), we considered that the reported results are reliable.   

*Results Section:*

- *For clarity please revise Figure 1 legends.*

**Our response:** Figure 1 and its legends are revised.

*Discussion section*

- *Please add a discussion about the quality of the char for combustion.*

**Our response:** Relevant information is provided in the discussion section.

# Peer review: Reviewer response for version 1

**DOI:** 10.21956/openreseurope.15435.r28088

**Published:** 2021-12-23

**Authors:** Luca Fiori, Donato Scrinzi

**Affiliations:** 1 Department of Civil, Environmental and Mechanical Engineering & C3A - Center Agriculture Food Environment, University of Trento, Trento, Italy; 2 Department of Civil, Environmental and Mechanical Engineering, University of Trento, Trento, Italy

The research article “Hydrothermal carbonization as an alternative sanitation technology: process optimization and development of low-cost reactor” is well written, and the research is meaningful and original. The authors cover many interesting aspects of HTC applied in a developing countries context for sanitation and struvite production from human wastes. It is definitely an interesting work, and we suggest it should be indexed after revision. The points here reported could help in that.

**INTRODUCTION.**

 Just a couple of suggestions.

1. “applying higher temperatures and longer retention times>.” We suggest replacing “retention times” with “reaction times”.
2. “A batch type reactor with 20 L capacity was constructed employing a relatively simple design and function at a material cost of around CHF 11K.” We suggest indicating costs in euros (or U.S. dollars) – at least in brackets.

**MATERIALS AND METHODS.**

**Human waste feedstock and hydrochar.**

1. The feedstocks used for HTC process were two stocks of faeces, which were dried and then re-moisturized to 20% dry matter. We wonder how much the initial moisture content in the untreated faeces is. The approach used by the authors is understandable and reasonable in a laboratory research context. However, the authors must mention that in the context of the practical realization of the concept, the raw material would be treated as it is, without preliminary drying. Having said that, as a matter of fact, the drying process may change some properties (like NH4-N content, dewaterability), thus the final properties of HTC products. We suggest mentioning this in the paper.
2. We suggest the authors explicitly write the extended form “relative centrifugal force” before the acronym RCF.

**Process optimization in commercial batch reactor**

1. The authors could consider explaining already in this part which are the tested HTC process temperatures and durations, after “several experimental conditions were tested.”
2. Moreover, the idea to test Touch & Down (TD) condition is very smart for low-cost applications without a heating controller, especially in the context of developing countries. Is there a particular reason for testing TD condition only for 200 °C and not for the other lower temperatures?
3. The naming for samples is not so straightforward. As a suggestion, it might be made clearer writing in full the Celsius degrees for reaction temperature and decades of minutes for duration, with TD considered as 0 minutes, e.g. instead of << Test-161 for HTC at 160 °C for 10 minutes and Test-206 for HTC at 200 °C for 60 minutes. The TD case, where the heating stopped when TR = 200 °C, is named Test-200.>> writing Test-160-10, Test 200-60, and Test-200-0, respectively.
4. We think the introduction of SF_adj is smart to make the severity factor more adherent to the real conditions and comparable. However, we found it difficult to understand the difference between SF vs SF_adj at a first reading. If we understood correctly reading Table 1, t_adj is defined as the time interval in which the reactor actually stays inside the range of ±3% of for the set temperature (and not a tolerance for time as I initially thought). We would suggest rephrasing the definition of t_adj in order to avoid any misconception.

 
**Material characterization**

1. Since it is crucial in the experimental procedure for ash production, the authors should specify at what temperature and residence time the muffle furnace worked for ash content analysis (for readers who do not have access to the standard DIN EN 14775). The same applies also for VS analyses (2540-E).

**Energy analysis**

1. There is some information useful for reproducibility about BMP tests which is missing. In particular, we suggest the authors expand this section with the substrate to inoculum ratio (SIR, referred to VS or COD) resulting from the mass chosen.
2. The use of thymolphthalein as pH indicator is interesting and it seems widely used in literature. As far as we can understand, this indicator can be used as a color signal for solution replacement (uncolored when pH < 9.3-10.5), in order to guarantee a proper CO _2_ removal. For the sake of reproducibility, the authors may add a reference or explain better how it can be useful. We found this research interesting ( https://doi.org/10.1002/tqem.21776) even if not fully explicative, but hopefully the authors may have better references to mention.
3. Before the process energy input for BLR tests, we suggest the authors mention how the energy input for tests with commercial reactor is considered/calculated (since it is shown in Table 2), and not only in the discussion.
4. Mg0 could be better explained as *fuel* gas bottle (since ‘gas’ can be confused with the aforementioned gas produced by BMP bottles, which could be mentioned as ‘biogas’ to avoid any misunderstanding).
5. “HV is the heating value of gas”: specify HHV or LHV.
6. “H _T_ is the enthalpy of saturated water at T.” In eq. (4) it is reported as H _r_: modify accordingly.
7. Consider to further explain the calculation of energy content of the HTC products (as far as we understand, it seems HHV_HC*mass_HC for solids and HHV_methane*mass_biomethane produced for process water).

**RESULTS**

**HTC of faeces**

1. “Much more pressure was produced in BLR-200 than in Test-200.” We suggest to rephrase: the gas is produced, not the pressure.
2. “and approached the maximum operational pressure.” Specify here in the main text that the pressure limit for BLR is 25 bar (data available in Extended Data)
3. We suggest to improve the quality of Figure 1: in terms of both graphics and legend: it is quite hard to follow the various curves in association with their relevant variables. Try to make it more immediate to read and understand.
4. Table 1 could be shown in the following page, closer to the results section.

**Energy contents**

1. Check the orthography: kJ, not KJ in capital letter (also in Table 2).
2. Table 2 (but valid in general), please explicitly mention in the units if the mass considered is on a dry basis or as received. The unit of total energy recovery ratio should be indicated as (%).
3. Total energy recovery ratio could be compared also to the energy that was present inside the initial feedstock (Table 2).
4. BMP results are slightly mentioned in the text and not compared with other works.
5. “The exothermic energy from the hydrothermal reaction of feedstock contributed to the total energy demand to a limited extent (< 4% of energy input).” Recent literature specifically dedicated to the evaluation of the exothermicity of the HTC reaction further corroborates this finding.

**Nutrient contents**

1. Table 1. Something is not right with respect to the data in table 1. The authors states that the “Oxygen contents estimated by subtracting the CHNS and ash fraction from 100 %.” But if we sum the CHNSO and ASH % we get values other than 100, beyond issues that can be related to rounding (e.g. for Faeces-1 we get 98.4). Check the correctness of all the data in Table 1.
2. Table 3. As a suggestion, the caption could be changed from ‘energy inputs’ to ‘Energy assessment’. Moreover, there must be an agreement between the convention on signs for endo- and exothermal reactions in both materials and method and results sections, e.g., changing the definition of equations (3) and (4) as Σ _i_(Energy) _i_=0 (all terms are positive if given to the system and negative if released by the system).
3. At the beginning of pg. 9, Table 5 can be explicitly named when referring to data of metals.
4. Table 4. ‘Loss from HTC’ does not seem the correct name, we’d rather say ‘Loss from experimental procedure/Loss after HTC’. The P mass balance should be closed (P mass is conservative).
5. The calculations for partitioning ratio in HC and PW are correct, but they can be explained in more detail in this section or, better, in the materials and methods section.

**Struvite precipitation**

1. The difference in the ratio among P:NH _4_:Mg could not be exactly the same for other phosphates precipitation, as indicated by the authors, but there could be also a not-statistically-significant difference inside the experimental error of the measurements (here missing).

**DISCUSSION**

**Scenario for energy and nutrient recovery**

1. Figure 3. Not all output streams are indicated (solid residues from leaching, liquid stream after struvite precipitation, etc). It can be useful to explicitly show them for further studies on recovery and environmental assessments of output waste streams. Moreover, for completeness’ sake, NH _4_-N initial value could be indicated also in the toilet/sanitation facility.

**Other general considerations.**

 We like the open approach by the journal and the authors’ decision to make available Extended Data with all the design detail of the reactor. Beyond the need to revise some aspects of the paper as highlighted above, the paper is of scientific interest and frames very well the problem of the management of human faeces in developing contexts that the authors propose to solve with HTC technology with basically simple reactors. Beyond the interesting experimental results, the authors analyse and discuss the advantages and limitations of the technology in the application context, from nutrient recovery to energy recovery.

 As a whole, a good paper that, after careful review by the authors, will deserve to be indexed.

Is the study design appropriate and does the work have academic merit?

Yes

Is the work clearly and accurately presented and does it cite the current literature?

Partly

If applicable, is the statistical analysis and its interpretation appropriate?

I cannot comment. A qualified statistician is required.

Are all the source data underlying the results available to ensure full reproducibility?

Yes

Are the conclusions drawn adequately supported by the results?

Yes

Are sufficient details of methods and analysis provided to allow replication by others?

Partly

Reviewer Expertise:

We (Luca Fiori and Donato Scrinzi) feel qualified to review and assess all the aspects of the paper.

We confirm that we have read this submission and believe that we have an appropriate level of expertise to confirm that it is of an acceptable scientific standard, however we have significant reservations, as outlined above.

## References

[1] : Comparison of two gas volume measurement systems by evaluating biochemical methane potential. *Environmental Quality Management* .2021; 10.1002/tqem.21776 10.1002/tqem.21776

# Response

**Published:** 2022-05-24

**Authors:** Jae Wook Chung

**Affiliations:** Zurich University of Applied Sciences, Switzerland

Dear Reviewers, We appreciate your comments. They were beneficial in improving this article, making it more precise and comprehensive. We accepted most of your comments and revised the manuscript accordingly. Please find our responses to the comments. We hope the second version of the manuscript addresses your comments sufficiently.

***Reviewer 1 / Comments***

*The research article “Hydrothermal carbonization as an alternative sanitation technology: process optimization and development of low-cost reactor” is well written, and the research is meaningful and original. The authors cover many interesting aspects of HTC applied in a developing countries context for sanitation and struvite production from human wastes. It is definitely an interesting work, and we suggest it should be indexed after revision. The points here reported could help in that.*

***INTRODUCTION.***

*Just a couple of suggestions.*

*“applying higher temperatures and longer retention times>.” We suggest replacing “retention times” with “reaction times”.*

**Our response:** The manuscript is revised accordingly (Introduction, paragraph 3)

*“A batch type reactor with 20 L capacity was constructed employing a relatively simple design and function at a material cost of around CHF 11K.” We suggest indicating costs in euros (or U.S. dollars) – at least in brackets.*

**Our response:** The cost is given in EUR (Introduction, paragraph 5).

***MATERIALS AND METHODS.***

***Human waste feedstock and hydrochar** **.***

*The feedstocks used for HTC process were two stocks of faeces, which were dried and then re-moisturized to 20% dry matter. We wonder how much the initial moisture content in the untreated faeces is. The approach used by the authors is understandable and reasonable in a laboratory research context. However, the authors must mention that in the context of the practical realization of the concept, the raw material would be treated as it is, without preliminary drying. Having said that, as a matter of fact, the drying process may change some properties (like NH4-N content, dewaterability), thus the final properties of HTC products. We suggest mentioning this in the paper.*

**Our response:** The primary consideration of the human waste experiments was assuring biosafety. Thus, all faecal stocks were autoclaved before introducing them into lab facilities. To provide a more explicit justification of the concept and limitation of this research, we provided more information on the feedstock and its preparation in the Methods section (Human waste feedstock and hydrochar). Also, we added the possible impact of feedstock pre-treatment in the discussion part (Positioning HTC in current sanitation technologies).

*We suggest the authors explicitly write the extended form “relative centrifugal force” before the acronym RCF.*

**Our response:** The manuscript is revised accordingly.

***Process optimization in commercial batch reactor***

*The authors could consider explaining already in this part which are the tested HTC process temperatures and durations, after “several experimental conditions were tested.”*

**Our response:** The manuscript is revised accordingly.

*Moreover, the idea to test Touch & Down (TD) condition is very smart for low-cost applications without a heating controller, especially in the context of developing countries. Is there a particular reason for testing TD condition only for 200 °C and not for the other lower temperatures?*

**Our response:** Testing more TD conditions in a lower temperature range would have provided valuable information for test conditions. However, it was limited by the amount of available feedstock, which was one of the main practical limitations. Based on the results from optimisation experiments, we expected that TD treatment in a lower temperature range (160-180 °C) would result in insufficient dewaterability of the resulting slurry from HTC treatment. The relevant information is provided in the Methods section (Process optimization in commercial batch reactor).

*The naming for samples is not so straightforward. As a suggestion, it might be made clearer writing in full the Celsius degrees for reaction temperature and decades of minutes for duration, with TD considered as 0 minutes, e.g. instead of << Test-161 for HTC at 160 °C for 10 minutes and Test-206 for HTC at 200 °C for 60 minutes. The TD case, where the heating stopped when TR = 200 °C, is named Test-200.>> writing Test-160-10, Test 200-60, and Test-200-0, respectively.*

**Our response:** The sample and Test IDs are revised as recommended throughout the manuscript.

*We think the introduction of SF_adj is smart to make the severity factor more adherent to the real conditions and comparable. However, we found it difficult to understand the difference between SF vs SF_adj at a first reading. If we understood correctly reading Table 1, t_adj is defined as the time interval in which the reactor actually stays inside the range of ±3% of for the set temperature (and not a tolerance for time as I initially thought). We would suggest rephrasing the definition of t_adj in order to avoid any misconception.*

**Our response:** The manuscript is revised accordingly.

***Material characterization***

*Since it is crucial in the experimental procedure for ash production, the authors should specify at what temperature and residence time the muffle furnace worked for ash content analysis (for readers who do not have access to the standard DIN EN 14775). The same applies also for VS analyses (2540-E).*

**Our response**: The relevant test conditions are provided.

***Energy analysis***

*There is some information useful for reproducibility about BMP tests which is missing. In particular, we suggest the authors expand this section with the substrate to inoculum ratio (SIR, referred to VS or COD) resulting from the mass chosen.*

**Our response:** VS-substrate (VSS) to VS-inoculum (VSI) ratio is provided.

*The use of thymolphthalein as pH indicator is interesting and it seems widely used in literature. As far as we can understand, this indicator can be used as a color signal for solution replacement (uncolored when pH < 9.3-10.5), in order to guarantee a proper CO _2_ removal. For the sake of reproducibility, the authors may add a reference or explain better how it can be useful. We found this research interesting ( https://doi.org/10.1002/tqem.21776) even if not fully explicative, but hopefully the authors may have better references to mention. (Konrad et al., 2021)*

**Our response:** We think that the use of thymolphthalein as a pH indicator is quite common practice in biogas laboratories. The relevant information is provided with the suggested reference.

*Before the process energy input for BLR tests, we suggest the authors mention how the energy input for tests with commercial reactor is considered/calculated (since it is shown in Table 2), and not only in the discussion.*

**Our response:** The relevant information is provided (at the end of Energy analysis section)

*Mg0 could be better explained as fuel gas bottle (since ‘gas’ can be confused with the aforementioned gas produced by BMP bottles, which could be mentioned as ‘biogas’ to avoid any misunderstanding)*

**Our response: **The manuscript is revised accordingly.

*“HV is the heating value of gas”: specify HHV or LHV.*

**Our response:** It is HHV. The manuscript is revised.

*“H _T_ is the enthalpy of saturated water at T.” In eq. (4) it is reported as H _r_: modify accordingly.*

**Our response:** The manuscript is revised accordingly.

Consider to further explain the calculation of energy content of the HTC products (as far as we understand, it seems HHV_HC*mass_HC for solids and HHV_methane*mass_biomethane produced for process water).

**Our response:** The manuscript is revised accordingly.  

***RESULTS***

***HTC of faeces***

*“Much more pressure was produced in BLR-200 than in Test-200.” We suggest to rephrase: the gas is produced, not the pressure.*

**Our response:** The manuscript is revised accordingly.

*“and approached the maximum operational pressure.” Specify here in the main text that the pressure limit for BLR is 25 bar (data available in Extended Data)*

**Our response:** The relevant information is provided both in the Methods (Bench-scale low-cost reactor) and Results section (HTC of faeces, paragraph 3). 

*We suggest to improve the quality of Figure 1: in terms of both graphics and legend: it is quite hard to follow the various curves in association with their relevant variables. Try to make it more immediate to read and understand.*

**Our response:** The graph and legend of Figure 1 are revised.

*Table 1 could be shown in the following page, closer to the results section.*

**Our response:** It is noted to the editorial team.

***Energy contents***

*Check the orthography: kJ, not KJ in capital letter (also in Table 2)*

**Our response**: The manuscript is revised accordingly.

*Table 2 (but valid in general), please explicitly mention in the units if the mass considered is on a dry basis or as received. The unit of total energy recovery ratio should be indicated as (%).*

**Our response:** The manuscript is revised accordingly.

*Total energy recovery ratio could be compared also to the energy that was present inside the initial feedstock (Table 2). **Our response:** The manuscript is revised accordingly (Results - Energy contents section and Table 2).*

*BMP results are slightly mentioned in the text and not compared with other works.*

**Our response:** The relevant information is added to the Discussion as a new paragraph. Also, the first part of the Discussion (Scenario for energy and nutrient recovery) is re-structured: an overview of the HTC process and resource recovery efficiency is provided. Then, energy recovery through solid fuel and biogas production is discussed in detail.

*“The exothermic energy from the hydrothermal reaction of feedstock contributed to the total energy demand to a limited extent (< 4% of energy input).” Recent literature specifically dedicated to the evaluation of the exothermicity of the HTC reaction further corroborates this finding*

**Our response:** We added the corresponding information to the Discussion part (Technical feasibility of low-cost reactor concept) with a reference (Ischia *et al.*, 2022).

***Nutrient contents***

*Table 1. Something is not right with respect to the data in table 1. The authors states that the “Oxygen contents estimated by subtracting the CHNS and ash fraction from 100 %.” But if we sum the CHNSO and ASH % we get values other than 100, beyond issues that can be related to rounding (e.g. for Faeces-1 we get 98.4). Check the correctness of all the data in Table 1.*

**Our response:** The contents of Table 1 are corrected.

*Table 3. As a suggestion, the caption could be changed from ‘energy inputs’ to ‘Energy assessment’. Moreover, there must be an agreement between the convention on signs for endo- and exothermal reactions in both materials and method and results sections, e.g., changing the definition of equations (3) and (4) as Σi(Energy)i=0 (all terms are positive if given to the system and negative if released by the system).*

**Our response:** The caption of Table 3 is revised as recommended. The signs of the energy terms now follow the convention of thermodynamics in both the material and methods and Table 3.

   

*At the beginning of pg. 9, Table 5 can be explicitly named when referring to data of metals.*

**Our response:** We would like to maintain the original manuscript. We consider it is more appropriate to present the data as nutrients because they also include non-metallic substances: P and NH4-N.

*Table 4. ‘Loss from HTC’ does not seem the correct name, we’d rather say ‘Loss from experimental procedure/Loss after HTC’. The P mass balance should be closed (P mass is conservative).*

**Our response:** The manuscript is revised accordingly.

*The calculations for partitioning ratio in HC and PW are correct, but they can be explained in more detail in this section or, better, in the materials and methods section.*

**Our response:** The relevant information is added in the Materials section (Material characterization).

***Struvite precipitation***

*The difference in the ratio among P:NH _4_:Mg could not be exactly the same for other phosphates precipitation, as indicated by the authors, but there could be also a not-statistically-significant difference inside the experimental error of the measurements (here missing).*

**Our response:** We agree that there might be insignificant experimental errors. However, we do not have enough tests to clear statistical insignificance. Instead, we think that the different analytical methods would result in the imbalance of P:NH4:Mg ratio. The manuscript is revised by adding this explanation. 

***DISCUSSION***

***Scenario for energy and nutrient recovery***

*Figure 3. Not all output streams are indicated (solid residues from leaching, liquid stream after struvite precipitation, etc). It can be useful to explicitly show them for further studies on recovery and environmental assessments of output waste streams. Moreover, for completeness’ sake, NH _4_-N initial value could be indicated also in the toilet/sanitation facility*.

**Our response:** We revised Figure 3 by adding nutrient contents: remaining amounts in the solid residue from acid leaching and out-solution from the precipitation process. However, NH _4_-N contents of solid samples were out of our research scope. Nutrient recovery was only considered after the combustion of hydrochar, and we assumed that ammonium content in HC was completely volatilised during the combustion of HC. Figure 3 and its caption are revised by providing more comments on NH4-N contents.
